# EcoBLMcrX, a classical modification-dependent restriction enzyme in *Escherichia coli* B: Characterization *in vivo* and *in vitro* with a new approach to cleavage site determination

**DOI:** 10.1371/journal.pone.0179853

**Published:** 2017-06-27

**Authors:** Alexey Fomenkov, Zhiyi Sun, Deborah K. Dila, Brian P. Anton, Richard J. Roberts, Elisabeth A. Raleigh

**Affiliations:** Research Department, New England Biolabs, Ipswich, MA, United States of America; Universität Stuttgart, GERMANY

## Abstract

Here we characterize the modification-dependent restriction enzyme (MDE) EcoBLMcrX *in vivo*, *in vitro* and in its genomic environment. MDE cleavage of modified DNAs protects prokaryote populations from lethal infection by bacteriophage with highly modified DNA, and also stabilizes lineages by reducing gene import when sparse modification occurs in the wrong context. The function and distribution of MDE families are thus important. Here we describe the properties of EcoBLMcrX, an enzyme of the *E*. *coli B* lineage, *in vivo* and *in vitro*. Restriction *in vivo* and the genome location of its gene, *ecoBLmcrX*, were determined during construction and sequencing of a B/K-12 hybrid, ER2566. In classical restriction literature, this B system was named *r*_*6*_ or *rglA*_*B*_. Like many genome defense functions, *ecoBLmcrX* is found within a genomic island, where gene content is variable among natural *E*. *coli* isolates. *In vitro*, EcoBLMcrX was compared with two related enzymes, BceYI and NhoI. All three degrade fully cytosine-modified phage DNA, as expected for EcoBLMcrX from classical T4 genetic data. A new method of characterizing MDE specificity was developed to better understand action on fully-modified targets such as the phage that provide major evolutionary pressure for MDE maintenance. These enzymes also cleave plasmids with ^m5^C in particular motifs, consistent with a role in lineage-stabilization. The recognition sites were characterized using a site-ranking approach that allows visualization of preferred cleavage sites when fully-modified substrates are digested. A technical constraint on the method is that ligation of one-nucleotide 5' extensions favors G:C over A:T approximately five-fold. Taking this bias into account, we conclude that EcoBLMcrX can cleave 3' to the modified base in the motif R^m5^C|. This is compatible with, but less specific than, the site reported by others. Highly-modified site contexts, such as those found in base-substituted virulent phages, are strongly preferred.

## Introduction

In recent years, two research areas have converged to stimulate interest in modification-dependent restriction enzymes (MDE): study of epigenetic phenomena, in which DNA base modifications modulate gene regulation and development (e.g., [1, 2]), and genetic manipulation of non-model bacterial species (e.g., [3–5]). At least 15 families of MDE have been discovered and characterized [6–8]. Some are used for study of base modification in eukaryotes (e.g. [2, 9–13]). These enzymes are the converse of conventional restriction-modification systems (RM), in which a sequence-specific endonuclease (REase) is paired with a protective modification methyltransferase (MTase). The spectrum of RM systems that can be maintained in a cell is limited by the specificity of resident MDE [14–16].

Four MDE have long been known in the independently isolated lineages *E*. *coli* K-12 and B. The *in vivo* specificities of three (*mcrA*_*K*_, *mcrBC and mrr*) have been characterized. Here we characterize the fourth MDE, EcoBLMcrX. The *ecoBLmcrX* gene was identified genetically during construction and analysis of the B/K-12 hybrid strain ER2566. ER2566 allows use of the favorable properties of the *E*. *coli* B strain BL21 while improving its response to stress, including MDE-MTase conflict. Suppression of the system formerly known as *mcrA*_*B*_ (*r*_*6*_, *rglA*_*B*_) was required. *r*_*6*_ was the earliest genetically-defined MDE. The present renaming of *ecoBLmcrX* (from *mcrA*_*B*_, *r*_*6*_, *rglA*_*B*_) distinguishes it from the K-12 *mcrA*_*K*_ *(rglA*_*K*_, *r6*_*K*_). Although this gene name diverges from the original convention [17], it follows that proposed for RM systems [18], which similarly vary among natural isolates within a species. Identification of *ecoBLmcrX* was accomplished by genome comparisons of ER2566 [19] with its cousin, BL21(DE3) [20] and with independent isolate *E*. *coli* K-12 MG1655. A B-specific genome island carries this fourth known MDE of the *E*. *coli* population, between *thiM* and *mrp*.

During our biochemical characterization of the enzyme in our laboratory, gene encoding this protein was studied based on its similarity to BisI using PsiBlast by another group of scientists [21]. We named the enzyme EcoBLMcrX to reflect biological role in counterpoint to conventional MTase-protected enzymes. It is grouped with other Type IV enzymes, which require at least one modified base for cleavage and typically display weak sequence selectivity.

A novel high-throughput sequencing approach was used to characterize *in vitro* cleavage of highly-modified DNAs by EcoBLMcrX and two related enzymes [8] from *Bacillus cereus* VD078 (Firmicutes) and *Nitrolancetus hollandicus* Lb (Green non-sulfur bacteria). The result is compared with site characterization by runoff sequencing from cleaved sites on low-complexity sparsely-modified DNAs. The reported recognition site for EcoBLMcrX was G^m5^C|NGC, using similar sparsely-modified DNAs [21]. With a larger sequence universe we find a weaker specification of the half-site, R^m5^C|, with activity highest in highly-modified regions. A complete specification of recognition sequence is hampered by probable systematic bias in favor of G or C 3' to the cleavage position, introduced by the sequencing method. Ligation of custom Illumina adaptors to a one-base 5' extension resulting from digestion likely over-represents fragments with G or C relative to those with A or T.

This work has added to our understanding of the variability of MDE, particularly in *E*. *coli*: there are at least four families, in three distinct genome-island locations. The relation between *in vivo* behavior and *in vitro* properties of this enzyme suggest that highly-modified phage are a primary target, while action at site-specific ^m5^C-modifications constrains distribution both of EcoBLMcrX-related genes and of sensitive MTases within the *E*. *coli* population.

## Material and methods

### Strain constructions and sequences

Strains, plasmids, genetic construction methods and details of sequence comparisons needed for characterization of EcoBLMcrX are described in S1 File EcoBLMcrX *in vivo*. The sequenced strain enabling EcoBLMcrX identification, ER2566, has additional useful features; its pedigree is displayed in S1 Fig. ER2566 lineage and in S1 Table ER2566 Construction Steps, Alleles and Sources.docx. These features of ER2566 and sequence interpretation are described in S2 File ER2566 Sequence Guide.

### Genome sequence analysis

Sequences of K-12 strain MG1655, B strain BL21(DE3) and ER2566 were aligned using the Geneious 7.1.9 implementation of Progressive Mauve [22, 23], as further described in S2 File. Annotation of variant positions was carried out using Genious. S2 Table 3way Comparison Annotations LCB1-5.xlsx contains 15 data fields for all annotations, one page per syntenic segment (Locally Colinear Block, LCB). S3 Table Recombination Patches and their Markers.xlsx extracts the limits of the recombination patches and constructed markers from S2 Table. S4 Table Recombination Patches Short Summary.xlsx presents accounting for gene numbers and DNA length in the patches.

### Enzymes and DNA manipulations

All REases, MTases, DNA polymerases, DNA and protein markers and the PURExpress *in vitro* transcription-translation system were from New England Biolabs (E6800; NEB, Ipswich, MA). PCR amplifications were performed using Q5 “Hot Start” DNA polymerase (M0493; NEB, Ipswich, MA). Oligonucleotides (Table 1) were from Integrated DNA Technologies (IDT, Coralville, IA). In Table 1, lower-case letters are homologous to the vector pSAP6 or pACYC; upper-case letters anneal to the genomic sequence.

**Table 1:** Primers.

Purpose	Sequence
pSAPV6:ECD_02033 forward PCR	tttaagaaggagatatacatATGCAAATGACAGAAACCCAGATC
pSAPV6:ECD_02033 reverse PCR	gggtcgacgaagagcggatccTCACAGCTTTGAGGTATCAAGCC
pSAPV6:ECD_02034 forward PCR	tttaagaaggagatatacatATGAGTGCACGTGAAGCATATC
pSAPV6:ECD_02034 reverse PCR	gggtcgacgaagagcggatccTTACACTGACTGAAACTCTTCAGTGAC
pSAPV6:ECD_02033_02034 forward PCR	tttaagaaggagatatacatATGCAAATGACAGAAACCCAGATC
pSAPV6:ECD_02033_02034 reverse PCR	gggtcgacgaagagcggatccTTACACTGACTGAAACTCTTCAGTGAC
pACYCDtet_NC_012971 mrp PCR	caggaaggtttaacatATGAACGAACAATCCCAGGCCAAATC
pACYCDtet_NC_012971 thiM PCR	gaggtgccgccggcttccattcaggatccTCATGCCTGCACCTCCTGC
primer S1248	TAATACGACTCACTATAGGG
primer S1271	TATGCTAGTTATTGCTCAG
primer pTetF	GTTGTAATTCTCATGTTTGACAGC
primer pTetR	GTTCTGCCAAGGGTTGGTTTGCGC
Illumina adaptor	5’_p_NAGATCGGAAGAGCACACGTCTGAACTCCAGTCdUACACTCTTTCCCTACACGACGCTCTTCCGATCT-3’

### MDE enzymes

Purified MDE enzymes NhoI and BceYI [8] were a generous gift of Shuang-yong Xu (NEB).

### Terminology

BL21(DE3) locus tags from Genbank NC_012971.2 are used as protein names until EcoBLMcrX is identified. ECD_02033 refers to current locus tag ECD_RS10565 or gene YP_003054628. This corresponds to multispecies record WP_001318412.1. ECD_02034 or EcoBLMcrX refer to current locus tag ECD_RS10570 or gene YP_003054629, designated *ecoBLmcrX* here. This corresponds to multispecies record WP_001276099.1.

All modified bases discussed are cytosines with additions at the 5 position. Particular modified sites will be represented as the top strand of reference sequences, with the modified base identified with a superscript: ^m5^C, ^hm5^C, ^ghm5^C. To indicate sequence-specific modification of both strands in a site, the top strand base that is paired with a modified bottom strand base will be underlined: C^m5^CGC. Degeneracies are represented as: W = A or T; S = G or C; R = A or G; Y = C or T.

### Identification, cloning and expression of the EcoBLMcrX modification-dependent restriction system

ER2566 contains a disrupted mini-Tn10 insertion mutation that abrogates restriction of T4*gt* (See S1 File and S2 File). Sequence comparison identified a disabled mini-Tn10-element within an *E*. *coli* B-specific genome island lying between conserved *thiM* and *mrp* genes (see S1 File for more detail and Results). Further localization employed primers listed in Table 1 to amplify the *thiM-mrp* locus containing the transposon disruption from ER2566 and from two drug-resistant insertion mutants, ER2490 and ER2491. The transposon insertions were located by restriction-mapping and amplicon sequencing, and found in CDS ECD_02033, upstream of CDS ECD_02034. These reading frames were PCR amplified from *E*.*coli* B strain BL21(DE) using primers of Table 1, and cloned separately and together into the T7 expression vector pSAPV6 [24] using the NEBuilder HiFi DNA Assembly Master Mix (E2621, NEB, Ipswich, MA). Both proteins were expressed *in vitro* using the PURExpress system (E6800, NEB, Ipswich, MA) and appropriate protein bands were detected on 10–20% SDS PAGE gel (S2 Fig Proteins from IVT Expression).

#### Modification dependence

Translation products were tested for endonuclease activity on DNAs that contained only unmodified C, ^5m^C, ^5hm^C, or ^Glu5hm^C modified phage DNAs: lambda (Dam^-^/Dcm^-^), Xp12, T4*gt* and T4 respectively. To further characterize EcoBLMcrX, the active enzyme was tested on a number of low-complexity substrates: *in vitro* modified pUC19 and pBR322 plasmid DNAs (using M.MspI (M0215), M.HpaII (M0214), M.HaeIII (M0224), M.HhaI (M0217), M.SssI (M0226) or M.CviPI (M0227) methyltransferases); or *in vivo* modified pBR322 carrying the *fnu4HIM* or *bbvIM* MTase genes. Digestion assays were performed in 50 μl NEB CutSmart buffer (B7204) with 1 μg of substrate DNA in the presence of RNaseA (10 ng/ul) at 37°C for 30–60 min.

#### High-throughput cleavage detection with high-density modification using MDEs EcoBLMcrX, BceYI and NhoI

Site identification using traditional runoff sequencing followed by oligonucleotide design is a laborious process. With identification of homologs BisI, BceYI and NhoI [8], and preliminary site characterization of the EcoBLMcrX (EcoBLI) enzyme by others [21], we designed experiments to take advantage of the expected one-base 5' extension using the Illumina high-throughput sequencing platform and a highly-modified substrate. With this approach we could characterize the recognition sites in a much larger sequence universe, using statistical bioinformatics approach.

In two of the substrates employed, modified C completely replaced C: phage DNAs Xp12 (^5m^C) and T4*gt* (^5hm^C). Densely modified plasmids pUC19 and pBR322 were methylated *in vitro* with M.SssI (^m5^CG) or M.CviPI (G^m5^C). More-sparsely modified substrates were clones of *fnu4HIM* (G^5m^CNGC) and *bbvIM* (G^m5^CAGC) cloned into pBR322, thus modified *in vivo*. These were digested with either EcoBLMcrX (produced in PURExpress) or the previously characterized MDE homologous to BisI: BceYI (expected to cleave GCNGC with 2–4 ^m5^C including the hemi-methylated site G^m5^CNG^m5^C) and NhoI (expected to cleave RCNGC with 3–4 ^m5^C [8]. Positive controls for the bioinformatics analysis were Fnu4HI (GCNGC)-digested unmodified pUC19 and pBR322. All these enzymes produce a one-base 5' extension.

Digestion products were ligated with custom sequencing adaptors (IDT): 5’-N extended-specific adaptors with full degeneracy at the 5' position (Table 1). The ligation mixture was purified on AxyPrep MAG PCR Cleanup beads (Axygen, CA), fractionated to 2 kb and PCR amplified for 15 cycles (NEB Next E6040). To enable analysis of 8 substrates digested with three different enzymes, 24 different index primers (product NEB Next ultra E7600, E7500) were used in separate amplifications. The substrate fragments and PCR libraries were quantitated using a 2100 Bioanalyzer (Agilent Technology, Santa Clara, CA). These 24 PCR libraries were then mixed to 4 nM and sequenced on a MiSeq sequencer (Illumina, CA) with a 2x100 bp paired-end reading protocol.

### Bioinformatic analysis of the recognition sequence from Illumina data

#### Read processing

Raw sequencing reads were first trimmed to remove adapter sequences and low quality bases at the 3’ end using the trim_galore program (Babraham Institute; http://www.bioinformatics.babraham.ac.uk/projects/trim_galore/). The trimmed sequences were then mapped to the reference genomes using the bowtie2 aligner allowing up to 1kb insert size (bowtie2—no-unal -X 1000) [25].) Those reads with high mapping quality scores (MAQ>20) were retained for further analysis.

#### Digestion site sequence definition

**Fig 1:**
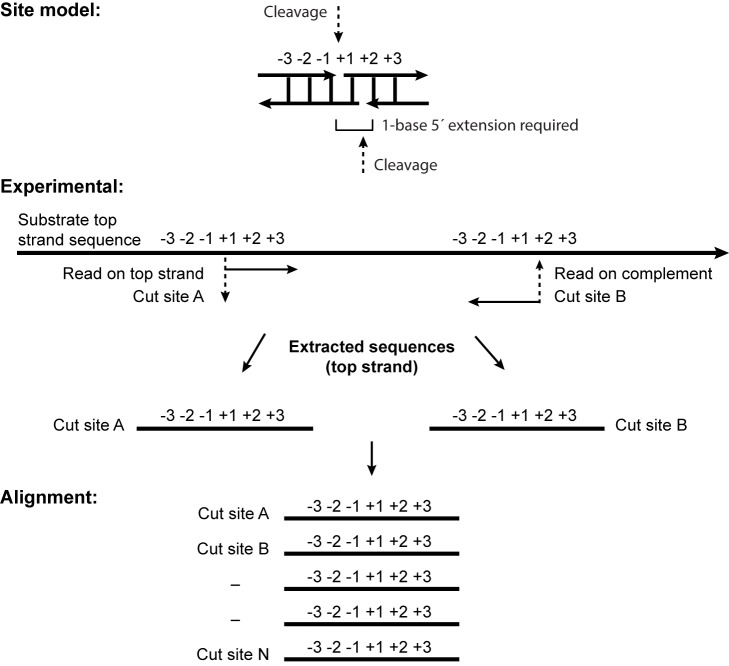
Illumina data analysis strategy.

Fig 1 shows the protocol for obtaining the putative cut sites. The 5' base of a read is a candidate digestion site and site of adaptor ligation. If the read matched the reference, we extracted the reference sequence at the ligation site plus 10 bases on either side—from -10 to +11 with respect to the 5' end in read A of Fig 1. If the read matched the complement, we extracted the reference sequence from -11 to +10 with respect to the 5' end of the read (read B of Fig 1). This definition accounts for the 1-base 5' extension assumed to result from cleavage. Any nicking events or double-strand cleavages with different configuration will not be detected by this analysis.

#### Data analysis by site ranking

The number of digestion events for a particular 5-base site formula was recovered as reads per site, normalized and ranked as described below. The ranked data were stored in an Excel database and were manipulated and displayed in table and graph form.

Dataset: For this analysis, the 21-base windows defined above were reduced to 5-base windows (-2 to +3 in Fig 1).

The following elements were computed for each enzyme:

Digestion site: each 5-base sequence represented in the dataset is defined to include both the site and (in parenthesis) its complement, with both strands presented 5'->3': n1n2n3n4n5 (n'5n'4n'3n'2n'1). For example, GCGAC (GTCGC). In this analysis, all the high-quality data can be used. All genomic loci that have the same 5-base sequence are combined and considered as one digestion site.

Freq digestion: A = Count of all reads aligning to a 5-base digestion site after trimming as above. Many particular loci along the substrate contribute to this frequency, since they are combined in defining the digestion site.

Freq substrate: B = Count of 5-base loci in the substrate genome that fit the site definition. Xp12 was the substrate presented here.

Freq normalized: C = A/B (Freq digestion/Freq substrate). This accounts for the fact that some 5-base sites are much more abundant in the substrate than others.

Relative Efficiency at site n: D = C (site n)/C (best site)

Number of mC: number of ^m5^C present, counting both strands. For Xp12, presented in Results, the number of ^m5^C residues = the number of C residues.

The data were collected in a database including: enzyme; for each enzyme: Digestion site sequence (with complement); values A-D; number of ^m5^C residues in the double-stranded site sequence.

## Results

### Mutant isolation and *in vivo* restriction of C-modified targets

Mutations that reduce restriction of C-modified DNA were isolated in the course of constructing ER2566, a strain related to BL21(DE3) (S1 Fig, S1 File, S1 Table). The rationale for its construction, the steps involved and resulting properties of this strain are detailed in S2 File ER2566 Sequence Guide, and in supporting tables (S2 Table, S3 Table, S4 Table). The sequence was reported in reference [19]. Briefly, it is a B/K-12 hybrid strain, with about 6% K-12-derived sequence replacing 7% of BL21 sequence.

Parental strains restrict entry of the ^hm5^C-containing phage T4*gt*, a phenotype called Rgl^+^, and are also unable to maintain some plasmid clones of ^m5^C site-specific methyltransferase genes, a phenotype called Mcr^+^. To obtain mutants, random transposon insertions were screened for Rgl^-^ (sensitivity to T6*gt*), rescreened for Rgl^-^ using T2, T2*gt* and T4*gt*, and tested for ability to maintain clones of methyltransferase genes that modify cytosine (the Mcr phenotype). Two of these mutants were pursued further, as described in S1 File.

### Identification of the EcoBLMcrX product and its gene

**Fig 2:**
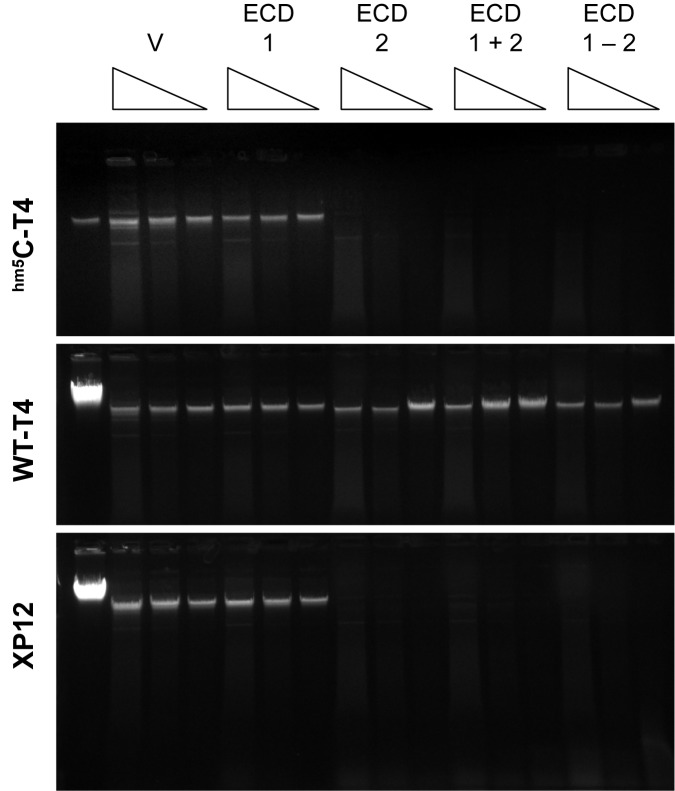
ECD_02034 is EcoBLMcrX. Products of *in vitro* translation (IVT) were incubated with three experimental substrates with complete base substitution of C. ^hm5^C-T4: T4*gt*, sensitive to restriction *in vivo*; WT-T4: wild type T4, glucosylated ^hm5^C, resistant *in vivo*; XP12: ^m5^C-substituted phage, as described in Materials and Methods. Triangles signify 3-fold dilutions of IVT products. V: IVT product of empty vector. ECD1: IVT product of ECD_02033, ECD2: IVT product of ECD_02034; ECD1+2: mixture of the separately translated products; ECD1-2: IVT with cotranscribed, cotranslated genes.

The translation products of adjacent coding sequences obtained as described in Materials and Methods were tested for activity on three phage DNA substrates: one protected from restriction *in vivo* (T4 wild type, containing glucosylated ^hm5^C), one sensitive *in vivo* (T4alpha*gt57* beta*gt14*, containing ^hm5^C; hereafter T4*gt*) and one candidate substrate (Xp12, an ^m5^C-substituted Xanthomonas phage) (Fig 2). Substrate disappearance was observed when and only when ECD_02034 protein (ECD2 in Fig 2) was present. Further dilution of ECD_02034 yielded a heterogeneous smear with sensitive substrates, consistent with endonucleolytic activity. Digestion of unmodified or Dam^-^Dcm^*+*^ lambda DNA resembled the lanes with vector alone (data not shown). This identifies ECD_02434 as EcoBLMcrX.

### EcoBLMcrX in vitro action depends on m5C modification context

To further characterize the specificity of the EcoBLMcrX enzyme, we used the IVT product to digest plasmid DNA substrates with distinct modification patterns. DNAs were initially free of all DNA modification (pBR322 produced in the methylation-deficient strain ER2796 [26]), then were modified *in vitro* with ^m5^C- producing MTases. DNA modified with M.AluI (AG^m5^CT), M.HaeIII (GG^m5^CC), M.HpaII (C^m5^CGG) or M.MspI (^m5^CCGG) did not produce a banding pattern (S3 Fig Modification patterns resistant to EcoBLMcrX).

**Fig 3:**
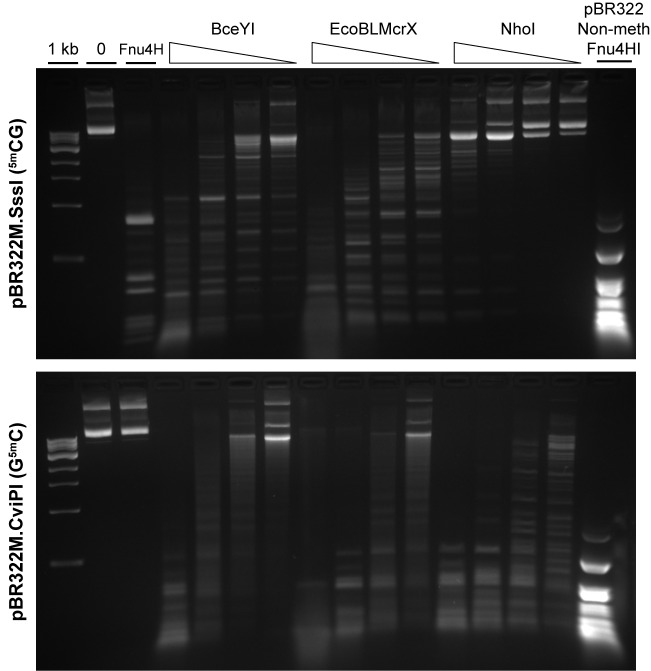
Three MDE action on densely modified targets. BceYI, EcoBLMcrX and NhoI MDE were tested in 3-fold serial dilution (left side of wedge = highest amount of enzyme) on pBR322 modified *in vitro* with (top panel) M.SssI (^m5^CG) or (bottom panel) M.CviPI (G^m5^C). 1kb: NEB 1 kb ladder. Controls: negative,— = no enzyme; Fnu4H: digested with the modification-protected enzyme Fnu4HI (GCNGC). Few of the 42 Fnu4HI sites in pBR322 overlap CpG sites, so Fnu4HI cuts extensively, but GpC modification protects fully. Positive control for Fnu4HI: pBR322 Non-Meth Fnu4HI = unmodified pBR322 digested with Fnu4HI.

In contrast, densely modified DNA, treated with M.SssI (^m5^CG) or M.CviPI (G^m5^C), yielded specific digestion patterns on a gel (Fig 3). Here we compared EcoBLMcrX with the recently-characterized MDEs [8] NhoI and BceYI. With ^m5^CG-modified DNA (upper panel), NhoI is distinct from the other two, with little activity. BceYI and EcoBLMcrX display similar size distribution during digestion but with distinct intermediate band patterns. For G^m5^C (lower panel), the three enzymes display similar size distributions. They all reach (leftmost lane for BceYI and NhoI) or pass through (EcoBLMcrX, next to left-most lane) digest patterns resembling the pattern produced by Fnu4HI (GCNGC) on unmodified plasmid (right-hand lane).

### High-throughput cleavage site analysis

The availability of the two MDE enzymes, NhoI and BceYI [8] together with the outline of the activity of apparently the same enzyme based on BLAST similarity [21] gave us confidence to attempt this comparison with a larger universe of cleavage positions. We took a high-throughput sequencing approach to identify recognition motifs that will yield a one-base 5' extension, allowing us to increase the sequence complexity of the analysis target. Custom Illumina adaptors with a 5'N instead of a 3'T were ligated with EcoBLMcrX, BceYI or NhoI- digested substrates as described in Materials and Methods. The resulting ligated DNA fragments were PCR amplified and quantified on the Bioanalyzer (S4 Fig Size-fractionated libraries for Illumina sequencing). The patterns displayed clearly show distinct cleavage patterns for the three MDE, particularly for substrates with ^m5^CG modification patterns (lanes 1–3 and 7–9 in S4 Fig).

#### WebLogo trials

To extract a site description from these data, WebLogo 3 analysis [27, 28] was tried first to identify and visualize over-represented motif sequences.

**Fig 4:**
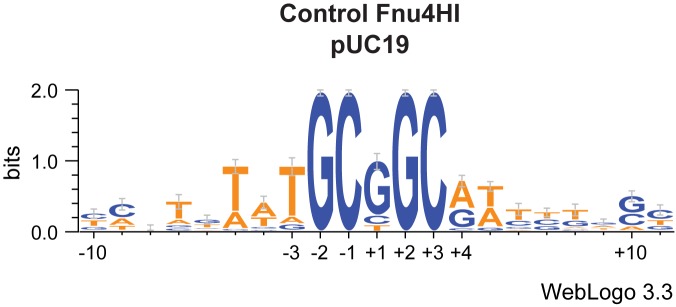
Control WebLogo—Type II enzyme on unmodified target.

**Fig 5:**
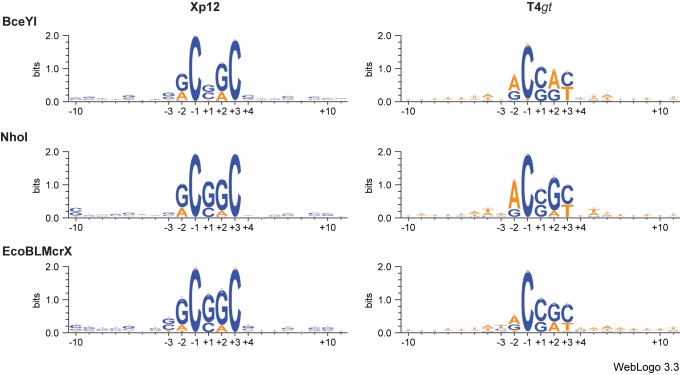
MDE WebLogos with C-replacement targets.

1. Control library. The analytic protocol was validated with a control library, made using a Type II enzyme with well-known properties. Fnu4HI cleaves unmodified GC|NGC, leaving a one-base 5' extension [29], with no bias at +1 (the N in the recognition motif). As expected (Fig 4), we found very strong signals for the 5' and 3' GC dinucleotides, with no degeneracy observed. Possibly because the substrate complexity is small (17 sites available; see further in (2)), the flanking nucleotides are also enriched for AT residues.

2. Bias at the ligation site. This library also alerted us to bias in recovery of the 5' extension. We observed enrichment for S (G or C) at +1 in the Fnu4HI control and all other libraries. We believe that those sites with G or C extensions are enriched in the libraries due to ligation bias during adaptor addition. The degree of bias is addressed below. The specific Fnu4HI sites present in the pUC19 substrate are slightly (9/17) enriched for AT extensions.

3. MDE digests of substituted phages. For these enzymes, the WebLogos (Fig 5) are compatible with but more relaxed than the site G^m5^CNG^m5^C proposed for the BisI family by Xu et al [8]. Importantly, most of the information determining cleavage is contained within the 5-base window surrounding the ligation site. When ^m5^C replaces C in Xp12 phage DNA, the recognition motif for BceYI may be summarized as RCNRC, where the -2 and +2 bases can be either G or A (Fig 5, top left panel). BceYI sites are slightly enriched in S (C or G) at +1, consistent with artifactual enrichment for S, selected during ligation.

For all three enzymes, a strong signal for C at -1, immediately adjacent to the position of cleavage, suggests that at least one of the two phosphodiester bond cleavages must be immediately 3' to ^m5^C. On the other hand, there is not a similarly strong requirement for G:^m5^C at +2. This is the position where the other strand cleavage must occur, since library preparation requires a one-base 5' extension. Potentially, the methyl group of T can serve instead of the methyl group of ^m5^C in the right surroundings. For the GlaI enzyme (recognition site G^m5^CG^m5^C; enzyme sequence is not available), full activity required the presence of four ^m5^C between the top and the bottom strand. However, 60% activity was displayed when either of the outer bases was substituted with an A:T or T:A base pair [30].

For NhoI and EcoBLMcrX, the motif is further enriched for S in the middle position, consistent with the suggestion of Xu et al [8] that NhoI depends more strongly on the number of ^m5^C in the site than do other members of the family.

All three MDE display a more relaxed specificity on the hydroxymethylated substrate (Fig 5, right hand three panels): RCNRY. The strongest signal is still the C at -1. Where Xp12 yielded a C at +3, either C or T is acceptable at +3 in T4*gt*. This may reflect the universe of available sites: T4 has a much higher AT content (62.7% AT) than Xp12 (30.9% AT). T4*gt* is particularly relevant, since it was the biological target used to identify the EcoBLMcrX enzyme.

4. WebLogo analysis of the remaining substrates is shown in S3 File Complete WebLogo Results. These displays agree that a five-base segment contains most of the information relevant to site determination. They do not lead to simple specification of a recognition site. In particular, they do not easily accommodate conditional site specification, in which what is present at one position (e.g. in one half-site) constrains what is accepted in another position.

#### Site ranking analysis

**Fig 6:**
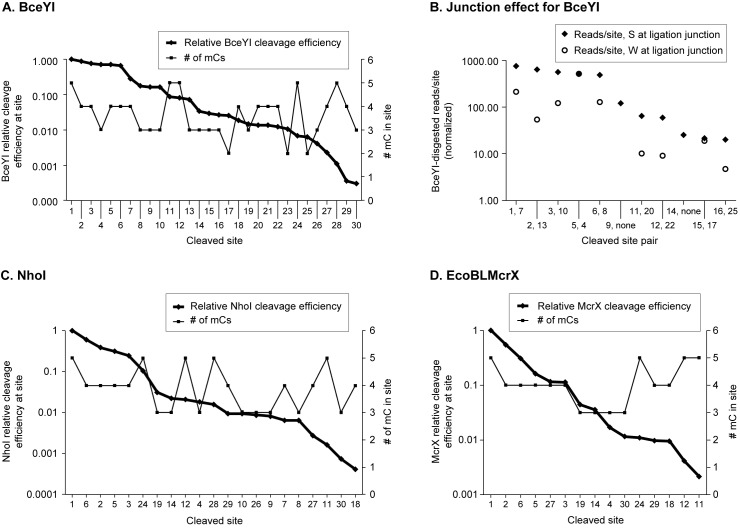
MDE site rank and ligation preference. On the X-axis, sites are numbered according to rank in BceYI digestion as in determined in S5 Table, and are listed in Table 2. On the Y-axis the normalized number of reads per site were ranked relative to the most-frequently-recovered site (Relative Efficiency, diamond symbols) as described in Materials and Methods for sites detected with at least 11 reads. The number of ^m5^C residues in both strands is also shown (square symbols, right-hand axis). Panel A, BceYI; panel C, NhoI; panel D, EcoBLMcrX. Panel B displays the effect of base composition at the adaptor ligation junction for BceYI. Sequence pairs (Table 3) that differ at the position of adaptor ligation (R = A or G; Y = T or C) were ordered according to Relative Efficiency as in panel A, and reads/site for S (R = G or Y = C; diamonds) and W (R = A or Y = T; crosses) were tabulated as paired values and displayed.

An advantage of the new approach taken here is that semiquantitative comparisons can be made both as between sites for a single enzyme and as between enzymes for a given site. For simplicity, we only describe results with Xp12, a ~46 kb substrate containing no ordinary cytosine, but only ^m5^C. As described in Materials and Methods, datasets for each of the three MDE were constructed with fields for five-base sequences containing double-strand cleavages, the number of reads aligning to examples of those sequences, and the number of examples of the sequences in the genome. The resulting data were compiled and ranked according to cleavage efficiency. The cleaved sites and their efficiencies (S5 Table Site Ranking Data 3 MDE Digests of Xp12.xlsx) are displayed graphically in Fig 6 and listed in Table 2. Each site has a unique number, reflecting the BceYI preference order; the same site numbering is used in Fig 6 for NhoI and EcoBLMcrX, but the order differs, reflecting cleavage preference. The effect of base composition at the ligation junction on measured reads/site is also shown in Fig 6 panel B for BceYI (from S6 Table Ligation junction effect); the site pairs are listed in Table 3.

**Table 2:** Site number assignments.

site(a)	BceYI (b) Fig 6 panel A		NhoI (b) Fig 6 panel C		McrX (b) Fig 6 panel D
1	GCGGC (GCCGC)	1	GCGGC (GCCGC)	1	GCGGC (GCCGC)
2	GCCAC (GTGGC)	6	ACGGC (GCCGT)	2	GCCAC (GTGGC)
3	GCGAC (GTCGC)	2	GCCAC (GTGGC)	6	ACGGC (GCCGT)
4	ACTGC (GCAGT)	5	ACCGC (GCGGT)	5	ACCGC (GCGGT)
5	ACCGC (GCGGT)	3	GCGAC (GTCGC)	27	GCCGA (TCGGC)
6	ACGGC (GCCGT)	24	GCCCC (GGGGC)	3	GCGAC (GTCGC)
7	GCAGC (GCTGC)	19	GTCAC (GTGAC)	19	GTCAC (GTGAC)
8	ACAGC (GCTGT)	14	GCCAT (ATGGC)	14	GCCAT (ATGGC)
9	ACCGT (ACGGT)	12	CCGGC (GCCGG)	4	ACTGC (GCAGT)
10	GCAAC (GTTGC)	4	ACTGC (GCAGT)	30	CAGTC (GACTG)
11	CCCGC (GCGGG)	28	GCGCC (GGCGC)	24	GCCCC (GGGGC)
12	CCGGC (GCCGG)	29	GCCTC (GAGGC)	29	GCCTC (GAGGC)
13	GCTAC (GTAGC)	10	GCAAC (GTTGC)	18	TCCGC (GCGGA)
14	GCCAT (ATGGC)	26	TAGCC (GGCTA)	12	CCGGC (GCCGG)
15	GCGAT (ATCGC)	9	ACCGT (ACGGT)	11	CCCGC (GCGGG)
16	ACCAC (GTGGT)	7	GCAGC (GCTGC)		
17	GCAAT (ATTGC)	8	ACAGC (GCTGT)		
18	TCCGC (GCGGA)	27	GCCGA (TCGGC)		
19	GTCAC (GTGAC)	11	CCCGC (GCGGG)		
20	CCTGC (GCAGG)	30	CAGTC (GACTG)		
21	CCCGT (ACGGG)	18	TCCGC (GCGGA)		
22	CCAGC (GCTGG)				
23	ACGAT (ATCGT)				
24	GCCCC (GGGGC)				
25	ACTAC (GTAGT)				
26	TAGCC (GGCTA)				
27	GCCGA (TCGGC)				
28	GCGCC (GGCGC)				
29	GCCTC (GAGGC)				
30	CAGTC (GACTG)				

a) number assignments were ordered using BceYI data; the same numbering was applied to the other enzymes.

b) 5-base sequences from the reference strand are shown, with the complement in parentheses. For each enzyme, the best sites are at the top, the worst sites at the bottom.

**Table 3:** Site pairs with GC vs AT in the central position.

Site pair	Sequence (complement)
1,7	GCRGC (GCYGC)
2,13	GCYAC (GTRGC)
3,10	GCRAC (GTYGC)
5,4	*ACYGC (GCRGT)
6,8	ACRGC (GCYGT)
9,none	†ACYGT (ACRGT)
11,20	CCYGC (GCRGG)
12,22	CCRGC (GCYGG)
14,none	†GCYAT (ATRGC)
15,17	*GCRAT (ATYGC)
16,25	ACYAC (GTRGT)

*pairs with anomalously high recovery of the W member.

†pairs for which the W member was not observed.

For BceYI, which had the deepest dataset, two points are evident. First, BceYI shows similar relative cleavage efficiency for its 6 best sites (panel A). This yields a high initial activity plateau, followed by stepwise efficiency drops. This behavior lends itself to summary as a motif with degeneracy, such as G^m^5CNG^m5^C. If all bases were equally acceptable in position +1 of this symmetric site, there should be two sites with the same efficiency, 1, each resulting from 50% of the reads (normalized to site occurrence). In fact there are six (not two) sites with relative efficiencies between 0.65 and 1, consistent with relaxation of the site specificity at an additional base(s). However, the bases at +1 (underlined below) are not the most variable ones (in bold): GCGGC (GCCGC), GCC**A**C (GTGGC), GC**G**AC (GTCGC), *ACTGC (GCAGT), *ACCGC (GCGGT), **A**CGGC (GCCGT).

Second, the composition of the base at the ligation junction (+1 in Fig 1) has a distinct effect on relative efficiency. In panel B, normalized reads/site for BceYI are displayed in pairs for sites with S (G or C) at +1 (diamonds) or W (A or T) (crosses) at the ligation junction. With exceptions (†,*), reads at W junctions were recovered about 20% as often as S junctions, in the same flanking sequence. The step-like pattern can still be discerned.

For two site pairs (*) S and W were recovered equally well (Table 3, Fig 6B). One of these pairs consists of two of the high-efficiency sites (starred above), while the other pair was recovered ~50-fold less often. For two other site pairs (†) no W junctions were recovered at all. We can't tell whether the anomalous recovery is due to effects on ligase action (e.g. from stacking effects) or to effects on enzyme recognition and digestion.

Table 4 summarizes the distribution of reads that identify the best six sites for the three enzymes (for complete data, see S5 Table and S6 Table).

**Table 4:** Comparing digestion by three MDE.

Enzyme	EcoBLMcrX	NhoI	BceYI
Best site	GCGGC (GCCGC)	GCGGC (GCCGC)	GCGGC (GCCGC)
Best site, fraction of normalized reads	0.42	0.36	0.17
Top 6 sites, fraction of normalized reads	0.94	0.94	0.8
Motif	RCSRC	RCSRC	RCNRY
Constraint proposed	4–5 mC	4–5 mC	3–5 mC, R = A allowed one occurrence
Anomalous site	GCCGA (TCGGC)	GCCCC (GGGGC)	ACTGC (GCAGT)
What's anomalous	Motif violated	Motif violated	Ligation bias violated
Fraction of normalized reads at anomalous site	0.05	0.02	0.03
Total reads	13286	720310	1345200
Sum normalized reads/site	29.92	1682	4433

All three enzymes recognize the same site as the "best site"—GCCGC (GCGGC), but this accounts for a greater fraction of normalized reads for EcoBLMcrX and NhoI than for BceYI. Similarly, the top six sites account for 94% of activity for EcoBLMcrX and NhoI but only 80% for BceYI. Five of the top six sites are same sites (in different orders) for the three enzymes, but for each enzyme one site in the top 6 is not shared with the other MDE. For BceYI, this is a site with T:A at the ligation junction (mentioned above); for the other two, the proposed motif is violated in different ways: missing the C at +3 for EcoBLMcrX, missing an R at +2 for NhoI.

## Discussion

### Genetic and genomic characterization of *ecoBLmcrX*, the classical restriction system *r6*, *rglA(B)*, *mcrA(B)*

The gene now designated *ecoBLmcrX* specifies this modification-dependent restriction activity. This is located in a genome island adjacent to *thiM* (S1 File Figure A). Two independent mutations isolated were insertions into ORF ECD_02033, an 81 codon ORF annotated as a transcription regulator, but the downstream ORF ECD_02034 was shown biochemically to code for the enzyme active *in vitro* (Fig 2). We infer that the insertions disrupt transcription of this downstream gene, with the resultant incomplete inactivation of the Mcr activity observed *in vivo* (S1 File).

### Three unrelated enzyme families restrict ^hm5^C-containing phages in *E*. *coli*

The EcoBLMcrX protein is unrelated to the K-12 MDE McrA, which belongs to the HNH family of nucleases. Instead it belongs to a family recently characterized by Xu et al. [8]. The distinction between *r*_*6*,*B*_ and *r*_*6*,*K-12*_ was already surmised from classical genetic mapping experiments [31] and confirmed by absence of hybridization of the K-12 *mcrA* (*r*_*6*, *K-12*_) to B DNA [31]. Although this activity was historically the first genetically-defined MDE, significant recent genetic and biochemical work [31–37] on the K-12 gene makes it undesirable to rename that gene. In contrast, K-12 and B share MDEs *mcrBC* and *mrr* [38].

### Bacteriophage T6*gt* defeats the activity of McrBC in K-12 and B, but does not defeat McrA or EcoBLMcrX

McrBC does not restrict T6*gt in vivo*, either in K-12 or in B [31], even though T6*gt* DNA is McrBC-sensitive *in vitro* [39]. Thus, T6*gt* is used specifically to test *in vivo* for McrA or EcoBLMcrX activity (aka *r*_*6*_) even in the presence of active McrBC (aka *r*_*2*,*4*_) [40]. The two *r*_*6*_ activities, *mcrA* (K-12) and *ecoBLmcrX* (B) are in fact very different, but both can act on T6*gt in vivo*. We emphasize here that the pattern of activity results from an uncharacterized *in vivo* interaction of McrBC with T6*gt*. *In vivo* protection from McrBC could result from an inhibitory activity imported with the phage DNA [41, 42] or from phage-induced activities expressed very early [43–46].

### Biochemical characterization of EcoBLMcrX

#### The product of ECD_02034 is necessary and sufficient to degrade DNA with ^m5^C or ^hm5^C

*In vitro* transcription-translation products (S2 Fig) were sufficiently free of contaminating activities to demonstrate that only one of the two proteins is required to degrade ^hm5^C containing T4*gt* DNA (Fig 2). When glucosylated, the DNA is not degraded, as expected from the lack of *in vivo* T4 wild-type phage restriction. The DNA of Xp12, which contains ^m5^C, is equally sensitive. Action on both ^m5^C and ^hm5^C is commonly observed for those MDE tested in vitro [7].

#### Densely C-modified DNA is degraded, while sparsely-modified DNAs are poor substrates for EcoBLMcrX and relatives NhoI and BceYI

A variety of modification MTases cognate to Type II restriction enzymes were tested and found not to yield EcoBLMcrX-sensitive substrates (S3 Fig). These yield sparsely-modified DNAs, with two ^m5^C on opposite strands in four-base sites. In contrast, two MTases with two-base sites yielded extensive digestion by EcoBLMcrX and relatives NhoI and BceYI (Fig 3). These three enzymes belong to a recently-described family in which at least some members require high-density modification [8].

#### Using high-throughput sequencing to compare three MDE: EcoBLMcrX, NhoI and BceYI

The availability of two related enzymes, NhoI and BceYI [8] together with the brief report of the enzyme activity [21] gave us confidence to attempt this comparison. Distinct banding patterns at the library preparation step (S4 Fig) presage distinct behavior in the sequencing results. Note that these patterns result only from cleavage events that yield a one-base 5' extension because of the requirement for adapter ligation and amplification. If digestion of each strand were independent, single-strand cleavages could result in double-strand cuts with a different extension, and we would not see these.

#### WebLogo visualization

Analysis of reads aligning to the substrate across 20-base windows (Fig 1) gave a collection of identified cleavage sites. Preferred cleavage sites were analyzed with WebLogo3 (S3 File Complete WebLogo Results). This tool is commonly used to visualize binding properties of sequence-specific DNA enzymes, such as transcription promoters and regulatory protein binding sites. As a control for the overall procedure, pBR322 was digested with the Type II restriction enzyme Fnu4HI, which cleaves GC|NGC, leaving a one-base 5' extension. The WebLogo result for this library shows some strengths and weaknesses of the method. The conserved GC motif elements of the five-base site emerge clearly, centered in the 20-base window with appropriate spacing (Fig 4). However, the central position (N) should display no preferred base; instead, there is enrichment for G or C. We attribute this enrichment to biased adaptor ligation efficiency during library preparation (see further below).

Weblogo visualization for the completely-substituted substrates Xp12 and T4*gt* (Fig 5) confirms that the relevant positions are within a 5-base window surrounding the position of cleavage, for all three MDE. The C at one cut site is highly conserved; the middle position again is spuriously enriched for G or C; the remaining positions are similar for the three enzymes but differ between the two substrates. This may reflect substrate GC content (63% vs 31%) rather than or in addition to the different modification (^m5^C or ^hm5^C).

#### A novel site-ranking approach to cleavage characterization

A novel visualization approach to the underlying data was tried, using a database of normalized, ranked data from the Xp12 libraries. Here, the 5-base sites are ordered according to frequency of appearance in the data, and cleavage+ligation efficiency displayed graphically (Fig 6). BceYI had the largest read database, and thus the deepest detection limit (see S5 Table and S6 Table). BceYI shows approximately equal preference for 6 sites (Fig 6 panel A), a property that favors motif definition of the kind familiar to users of Type II restriction enzymes. For this enzyme, the motif RCNRY (when including at least 3 ^m5^C) includes all the favored sites, although a T must be mediating cleavage on one strand for two of these (GCCAC (GTGGC) and GCGAC (GTCGC), the second and third best sites). Sites that fit the motif but were not detected include primarily those with only two ^m5^C. The presence of a second plateau in the distribution of reads per site (Fig 6 panels A, B) suggests that a set of secondary sites (similar to "star activity" (see, e.g. [47]) may be specifically recognized with lower efficiency. The buffer used was not the optimal BceYI buffer (S.Y. Xu, personal communication).

In contrast, EcoBLMcrX and NhoI did not cleave a set of sites with similar preference (Fig 6 panel C and D), but instead showed a declining preference over various site configurations. Each of the three enzymes favored five of the same six sites, but each displayed a preference for a distinct sixth site (Table 4; S5 Table Site Ranking Data 3 MDE digests of Xp12.xlsx).

#### A/T pairs are selectively lost in ligating 5' 1-nucleotide extensions in this experiment

The effect of base composition at the ligation site (Fig 4, Fig 6 panel B) strongly suggests that the ligation efficiency of the one-base 5' extension is distorting the ranking of sites. The characterized Type II restriction enzyme Fnu4HI showed this clearly (Fig 4). The MDE BceYI data (Fig 6 Panel B) suggest that the magnitude of the effect is 3- to 5-fold; G/C is more highly represented than the same site with A/T at +1. Some particular site pairs did not show this discrepancy, which could reflect the action of either the cleaving enzyme or the ligation enzyme. Accordingly, only general trends can be confidently described.

### EcoBLMcrX recognition site

EcoBLMcrX recognizes and cleaves sites of the form RCSRC when 4 or 5 ^m5^C residues are present (Table 4). However, this description is likely not the whole answer, for two reasons. First, such sites should be rare in the biological target, T4*gt*, which has a low GC content (62.7% AT), but T4*gt* is a good substrate *in vitro* (Fig 2; S4 Fig lane 16). Second, the protocol used will detect only those sites with a 5' 1-base extension, so the description above results only from those fragments. A notable aspect of the EcoBLMcrX libraries was the poor read depth obtained (Table 4); only 10% as many reads were recovered for Xp12 digested with EcoBLMcrX as for BceYI. Three factors could contribute to this. First, impurities in the enzyme preparation could result in loss of the 5' extensions, although negative control substrates (Fig 2, T4 wt; S3 Fig, lane non-modified pBR322) are not noticeably degraded. Second, variable amounts of the different libraries may have mixed in the sequenced pool due to quantitation inaccuracies. Third, the enzyme might act independently on the two strands, leaving a variety of extensions at fragment ends. The steadily declining Relative Cleavage Efficiency (Fig 6 Panel D) could result from the interaction of factors: the linear distribution of different sites on the genome, the relative efficiency of cleavage at them, and the ligation efficiency of the extensions thus produced. In that case, the site might be represented as R^m5^C| where N = G,C>AT.

### Bacteriophage as biological drivers of MDE *in vitro* properties

A phage-defense role for MDE can explain the weak sequence specificity often observed for MDE. Members of the present class of MDE (EcoBLMcrX, NhoI, even BceYI) depend on heavy modification for efficient cleavage. Heavily modified DNA of this sort has only been found in virulent phages thus far. When acting on phage DNAs with full substitution of C by ^m5^C or ^hm^5C, MDE don't need a highly specific site—cleavage at all modified positions would be sufficient. In contrast, ordinary restriction enzymes are constrained to maintain the same specificity as the cognate MTase to avoid killing the host cell. Some sequence-specificity in the MDE is needed to allow coexistence with solo housekeeping MTases (such as Dam), and with the M activities of the host's other RM systems. For this purpose, specification of one additional base appears to be sufficient.

Such weakly-specific MDE will still play a role in management of foreign DNA that does not come from virulent phage. For action on other substrates—temperate phages, plasmids or foreign chromosomal DNA—many distinct site-specific modification patterns are likely to be encountered. A relaxed MDE specificity like that observed will also allow attack on diverse patterns. Recognition of a highly-specific modified sequence will be reserved for special situations where selection is imposed for specific recognition of flanking bases. The GATC/Gm6ATC/GATm4C,cassette exchange system in in *Streptococcus pneumoniae* imposes such a selection (see, e.g. reference [48]).

## Supporting information

S1 File EcoBLMcrX *in vivo*. (PDF)

S2 File ER2566 sequence guide. (PDF)

S3 File Complete WebLogo results. (PDF)

S1 Fig ER2566 lineage. Abbreviations: td, transduction; TetS, tetracycline sensitive. ER2566 carries no drug resistances. See S2 File for description of rearrangement nomenclature, S1 Table Part A for assignment of recombination patches to syntenic segments (LCB) and Part B for allele sources.
(EPS)

S2 Fig Proteins from IVT expression. PURExpress *in vitro* transcription-translation products were Control_vector: pSAPv6; ECD_0233: pSAPv6_ECD_0233 (9.1kD protein); ECD_0234 (EcoBLMcrX): pSAPv6_ECD_0234 (15.9 kD protein); ECD_02033+02034(EcoBLMcrX): a mixture of the two products; or ECD_0233_0234 (EcoBLMcrX): one reaction from the operon arrangement, pSAPV6_ECD_0233_0234 (EcoBLMcrX). Background bands present in all lanes are translation factors and residual ribosomal proteins.
(EPS)

S3 Fig Modification patterns resistant to EcoBLMcrX. pBR322 was methylated *in vitro* as indicated then digested with IVT products of the pSAPv6 vector alone (-) or the clone of ECD_02034 (+). Non-modified pBR322 (none) is the negative control; M.SssI (^m5^CG) is the positive control.
(EPS)

S4 Fig Size-fractionated libraries for Illumina sequencing. Agilent Bioanalyzer display of amplified digests. Substrates (mol) with different modification patterns (Mod) were digested (Enz), ligated, PCR amplified and sized on the Bioanalyzer. The products that migrate at or below 1000 nt were used for sequencing reads. Mol (substrates): pUC19 (pU); pBR322 (pB); Xp12; T4*gt*; p*fnuM* = pBR322*fnu4HIM*; p*bbvIM* = pBR322*bbvIM*. Mod: modification pattern; MG, ^m5^CG (with M.SssI); GM, G^m5^C (with M.CviPI); ^m5^C all C residues; ^hm5^C all C residues; G^m5^CNGC; G^m5^CWGC; none, no modification. Enz: M, EcoBLMcrX (1, 4, 7, 10, 13,16, 19, 22); N, NhoI (2, 5, 8, 11, 14, 17, 20); B, BceYI (3, 6, 9, 12, 15, 18, 21), F, Fnu4HI (23, 24).
(EPS)

S1 Table ER2566 construction steps. (PDF)

S2 Table 3way comparison annotations LCB1-5. (XLSX)

S3 Table Recombination patches and their markers. (XLSX)

S4 Table Recombination patches short summary. (XLSX)

S5 Table Site ranking data 3 MDE digests of Xp12. (XLSX)

S6 Table Ligation junction effect. (XLSX)
