# Supplementary material for: EcoBLMcrX, a classical modification-dependent restriction enzyme in Escherichia coli B: Characterization in vivo and in vitro with a new approach to cleavage site determination
Source: PLoS One. 2017 Jun 27;12(6):e0179853. doi: 10.1371/journal.pone.0179853 (PMC5487053; doi:10.1371/journal.pone.0179853)
Supplement: S1 File — (PDF) [file pone.0179853.s001.pdf]

Characterization of EcoBLMcrX *in vivo*: bacterial and bacteriophage strains and constructions

Deborah Dila, Alexey Fomenkov and Elisabeth A. Raleigh

Supplement to: EcoBLMcrX, a Classical Modification-Dependent Restriction Enzyme in *Escherichia coli* B: Characterization *in vivo* and *in vitro* with a New Approach to Approach to Cleavage Site Determination

Alexey Fomenkov, Zhiyi Sun, Deborah K. Dila, Brian P. Anton, Richard J. Roberts, Elisabeth A. Raleigh.

Materials and Methods

The bacterial strains, plasmids and phages used in this study are listed in Tables 1-2. Defective Tn10 transposons Tn10dTet (element 104) or Tn10dCm (element 105) [1] are also known as miniTn10 elements, and appear as mTn10 TcR or mTn10 CmR in Figure A. ER2683 (Table 2) was used for plasmid constructions.

<u>Media and reagents</u>

Media for constructions and restriction tests were as in reference [5]; for cloning and expression, LB broth or agar with appropriate antibiotics was used.

<u>Genetic isolation of *mcr*-73 *and mcr-74 E. coli* B mutants</u>. Briefly, starting strains lacked the other *E. coli B* restriction systems (ER2384 or ER2467; Table 1). Libraries of random Tn10dTet or Tn10dCm transposon insertions [1] were screened for nibbled colonies on plates spread with T6*gt* (the Rgl$^-$ phenotype; [6]). These were purified, rescreened with T4*gt*, and then tested for sensitivity to T2, T2*gt*, T4*gt*, and T6*gt*. Each candidate was backcrossed using P1*vir* [7] to its parent and/or grandparent and against each other.

Table 1. Bacterial Strains Used for Characterization of EcoBLMcrX *in vivo*

***E. coli* strains**	**Genotype**	**Source**
BL21 (DE3)	*E. coli* B *hsdS gal ompT* [Lon^{-}] [Dcm^{-}] [DE3=λ *sBamH1o ΔEcoRI-B int::lacI::PlacUV5::T7 gene1 i21 Δ nin5*]	Studier [2]
ER2384	*IN(IS1_26-Δ(yjiT-opgB)114::IS10-IS1_1) [plon::IS186] del[aaad-ompT-cusS] del(galK-yghJ) uvrY::IS1::del(yecF-dcm-hchA)*	This work
ER2431	*ER2384 mcr-72, zzz::mTn10Cm*	This work
ER2467	*IN(IS1_26-Δ(yjiT-opgB)114::IS10-IS1_1) fhuA2::IS2 [plon::IS186] del[aaad-ompT-cusS] del(galK-yghJ) psulA211(-37 T->C) uvrY::IS1::del(yecF-dcm-hchA)*	This work
ER2475	ER2467 *mcr-73::mTn10Tet*	This work
ER2477	ER2467 *mcr-74::mTn10Cm*	This work
ER2490	ER2384 *mcr-74::mTn10Cm*	This work
ER2491	ER2467 *mcr-74::mTn10Cm*	This work
ER2566	*IN(IS1_26-Δ(yjiT-opgB)114::IS10-IS1_1) fhuA2::IS2 lacZ::T7gene1 [plon::IS186] del[aaad-ompT-cusS] del(galK-yghJ) psulA211(-37 T->C) uvrY::IS1::del(yecF-dcm-hchA) mcr-73::(mTn10 tetA::IS10) IN(speA210::IS10-xanA::IS10) endA1(E208K)*	This work; see S2 File for the remainder of its construction
ER2683	F128 (ER2250) *proA^{+}B^{+} lacIq Δ(lacZ)M15 zzf:: miniTn10(KanR)/ fhuA2::IS2 Δ(lacI-lacA)200 glnX44 e14- rfbD1? relA1? endA1 spoT1? thi-1 Δ(yjiT-opgB)114::IS10*	Raleigh E. (NEB)

Two candidates gave Rgl- phenotypes genetically linked to the transposon drug resistance phenotypes. These were tested for their Mcr phenotype (restriction of m5C-containing DNA) by transformation with MTase plasmids: these expressed M.SssI (m5CG), M.BbvI (Gm5CWGC), M.HpaII (Cm5CGG) and M.HgaI (Table 2). A tetracycline-sensitive derivative of the TetR mcr-73::Tn10dTet insertion (described further below) was isolated using the procedure of Maloy and Nunn [8]. A more-complete description of the pedigree of ER2566 and its properties may be found in the

supplemental files: S1 Figure ER2566 lineage, S2 File T7 Express sequence guide, S1 Table 3way comparison annotations LCB1-5, S2 Table Recombination Patches and their Markers, S3 Table Recombination patches short summary.

Table 2. Bacteriophage Strains and Plasmids

**Name**	**Properties**	**Source**	**Reference**
Xp12	Wild type; 5mC modified phage	Melanie Ehrlich	[3]
T2H	Wild type (Hershey)	Helen Revel	[4]
T2*gt2*	^{5hm}C modified phage	Helen Revel	[4]
T6*gt41*	^{5hm}C modified phage	Helen Revel	[4]
T4gt=T4α*gt57* β*gt14*	^{5hm}C modified phage	Helen Revel	[4]
P1vir	transducing phage	N. Kleckner	
λNK1323	Element 104 transposon donor	N. Kleckner	[1]
λNK1324	Element 105 transposon donor	N. Kleckner	[1]
pSAPV6	p15 replicon with a T7 expression cassette	Morgan R. (NEB)	
pSAPV6:ECD_02033	ECD_02033 expression vector	This work	
pSAPV6:ECD_02034	ECD_02034 expression vector	This work	
pSAPV6:ECD_02033_02034	ECD_02033-02034 expression vector	This work	
pACYCDtet_NC_012971	constitutive expression vector with *thiM-mrp* fragment from *mcr-73* host	This work	
pBR322*fnu4HIM*	M.Fnu4HI methylase expression vector	Xu Y.-S. (NEB)	
pBR322*bbvIM*	M.BbvI methyltransferase expression vector	Xu Y.-S. (NEB)	
p*sssIM*	M.SssI methyltransferase expression vector pCAL7	W. Jack (NEB)	
p*hpaIIM*	M.HpaII methyltransferase expression vector	G. Wilson (NEB)	
p*hgaIM*	M.HgaI methyltransferase expression vector	G. Wilson (NEB)	

Results

Genetic and phenotypic properties of three mutants

Of three insertion mutations analyzed, two were genetically linked to each other and to the Rgl- phenotype. They displayed distinct Mcr phenotypes. For the third, the Rgl- phenotype was not linked to the drug resistant insertion, and the insertion was not linked to the other two insertions.

1. *mcr-73::Tn10dTet* in ER2475 showed 100% linkage of TetR with Rgl$^-$ (12/12 in each of 2 crosses). This mutant was transformable with pCAL7 and p*bbVIM*; when backcrossed to the parent, yielding ER2479, it was again permissive for pCAL7 and p*bbVIM* but not p*hpaIIM*. This phenotype was also displayed by TetS derivatives of 2 independent backcrossed strains.
2. *mcr-74::Tn10dCm* in ER2477 showed 100% linkage to Rgl$^-$, yielding the backcross strains ER2490, ER2491. These were transformable with pCAL7 but not p*bbVIM*. It was more permissive for pCAL7 than its parent but not as much as the *mcr-73* line, and showed no improvement for p*bbvIM*I or p*hpaIIM*. As described further below, the transposon inserted at the same 9-bp site as that of *mcr-73*, consistent with the known site-preference of Tn10 [9].
3. *mcr-72, zzz::Tn10dCm* in ER2431 carries an insertion that is not linked to the phenotype or the other two insertions. Transduction of TetR from ER2475 (*mcr*-73) into ER2431 yielded TetR CamR, so the *mcr*-73::*Tn10dTet* is unlinked to *zzz::Tn10dCm*. The phenotype of *mcr-72* includes improved acceptance of p*bbVIM*, ambiguous improvement for pCAL7, but reduced acceptance of p*hpaIIM*. We propose that the insertion occurred in a cell already carrying a spontaneous

mutation in one or more of the genes identified here. Spontaneous mutations described earlier using the same strategy [6] always required vitamin B1, and were probably deletions mediated by a nearby IS1 extending to *thiM*.

<u>Position of *mcr-73* and *mcr-74* transposon insertions:</u>

We compared the ER2566 sequence of the region encoding EcoBLMcrX with those of B and K-12 strains, with Tn10 and with the IS10 components of Tn10 (Figure A). The insertion is found in a genome island (a region with different content in different isolates of *E. coli*) that lies between shared genes *thiM* and *mrp*. The version of the island in B strains includes seven hypothetical genes and an IS1 element, while the corresponding K-12 region contains seven genes unrelated to those in B. The *mcr-73* Tn10dTet and *mcr-74* Tn10dCm have both inserted into the ECD_02033 ORF of *E.coli* B BL21(DE3) in this island; in ER2566, an additional IS10 has inserted into the TetA gene within the transposon, resulting in loss of tetracycline resistance.

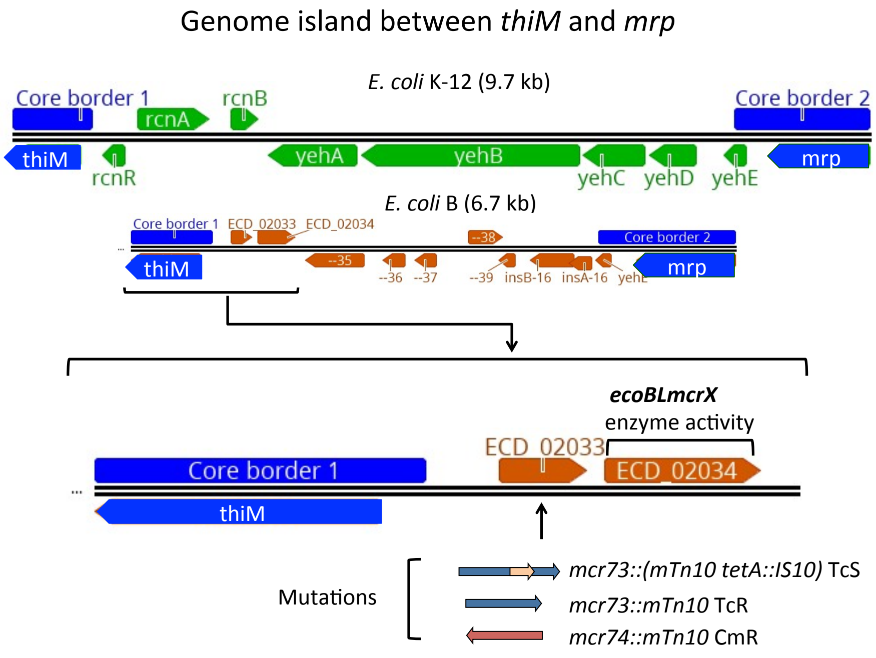

Figure A. EcoBLMcrX genome island context. From the top to bottom are shown the K-

12 and B genome islands at *thiM*, and enlargement of the BL21(DE3) region coding for EcoBLMcrX. The region between conserved flanking genes *thiM* and *mrp* is nonhomologous between MG1655 and BL21(DE3). EcoBLMcrX is encoded at the left end of the BL21(DE3) region. Insertion mutations that alter its expression in vivo disrupt an adjacent gene. Tapered boxes are CDSs (blue, conserved genes; green, *E. coli* K-12-specific; orange, *E. coli B* specific). Blue, red and yellow boxes with arrowheads are transposable elements mTn10 or IS10, oriented according to transcription of the cargo drug resistance gene (TcR, CmR) or transposase (IS10) (transposons not to scale).

PCR amplification, cloning and sequence analysis of the genome environment of insertion mutations *mcr-73* and *mcr-74* showed that both are located at the same site in the ECD_02033 ORF. Tn10 is known to insert preferentially at favored sites [10], so recovery of two independent insertions at the same site (Figure B) is unsurprising.

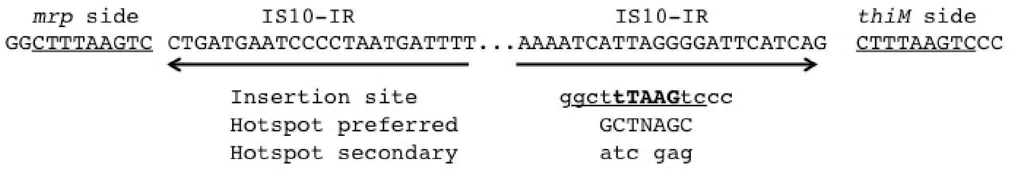

Figure B. Comparing the mTn10 insertion site to known hot sites. Tn10 insertion at a 9 bp target (underlined, *mrp* side) creates a second copy on the other side of the insertion (underlined, *thiM* side), flanking inverted repeats from IS10s (IS10-IR; arrows). Below the diagram, the original target site is aligned with nucleotides found at insertion hotspots: highly preferred (Bold Caps) or enriched (bold lowercase). Bases in agreement with this consensus are highlighted in red. The site as shown is in the reverse orientation from that shown in Figure A to facilitate comparison with Tn10 literature.

Both insertions *mcr-73::Tn10dTet* and *mcr-74::Tn10dCm* generate the same local structure but carry different cargo genes differently oriented. In *mcr-73,* transcription from *tetRp* within the transposon could potentially lead to readthrough expression of ECD_02034 (Figure A). In *mcr-74,* transcription from *catp* would be in the opposite orientation, away from ECD_02034 (Figure A), potentially explaining the phenotypic differences between the two in magnitude of Mcr restriction described above.

Tolerance of pCAL7, ^{m5}CG by the mutant *mcr-73* in contrast to the parent suggested that the enzyme might attack ^{m5}CG in general, e.g. M.HpaII-modified substrates. Instead, ^{m5}CG-modified DNA is a poor substrate, and M.HpaII-modified substrates are not digested at all (main text). Thus, inability of parent or mutants to tolerate p*hpaIIM* (1 above), must result from some other property of the p*hpaIIM* plasmid.

References

1. Kleckner N, Bender J, Gottesman S. Uses of transposons with emphasis on Tn10. Methods Enzymol. 1991;204:139-80. doi: 10.1016/0076-6879(91)04009-D. PubMed PMID: 1658561.

2. William Studier F, Rosenberg AH, Dunn JJ, Dubendorff JW. [6] Use of T7 RNA polymerase to direct expression of cloned genes. Meth Enzymol. 1990;185:60-89. doi: 10.1016/0076-6879(90)85008-c.

3. Kuo TT, Huang TC, Wu RY, Chen CP. Phage Xp12 of Xanthomonas oryzae (Uyeda et Ishiyama) Dowson. Can J Microbiol. 1968;14(10):1139-42. PubMed PMID: 5681525.

4. Georgopoulos CP, Revel HR. Studies with glucosyl transferase mutants of the T-even bacteriophages. Virology. 1971;44(2):271-85. PubMed PMID: 4935677.

5. Kelleher JE, Raleigh EA. Response to UV damage by four Escherichia coli K-12 restriction systems. J Bacteriol. 1994;176(19):5888-96. Epub 1994/10/01. PubMed PMID: 7928948; PubMed Central PMCID: PMCPMC196804.

6. Revel HR. Restriction of nonglucosylated T-even bacteriophage: properties of permissive mutants of Escherichia coli B and K12. Virology. 1967;31(4):688-701. Epub 1967/04/01. PubMed PMID: 4290282.

7. Sternberg NL, Maurer R. Bacteriophage-mediated generalized transduction in Escherichia coli and Salmonella typhimurium. Methods Enzymol. 1991;204:18-43. doi: http://dx.doi.org/10.1016/0076-6879(91)04004-8. PubMed PMID: 1943777.

8. Maloy SR, Nunn WD. Selection for loss of tetracycline resistance by *Escherichia coli*. J Bacteriol. 1981;145(2):1110-1. PubMed PMID: 7007341; PubMed Central PMCID: PMCPMC217228.

9. Kleckner N. Transposon Tn10. In: Berg DEaH, M. M. , editor. Mobile DNA. Washington, D.C.: American Society for Microbiology; 1989. p. 227–68.

10. Bender J, Kleckner N. Tn10 insertion specificity is strongly dependent upon sequences immediately adjacent to the target-site consensus sequence. Proc Natl Acad Sci USA. 1992;89(17):7996-8000. PubMed PMID: 1325639; PubMed Central PMCID: PMCPMC49842.
