# Supplementary material for: EcoBLMcrX, a classical modification-dependent restriction enzyme in Escherichia coli B: Characterization in vivo and in vitro with a new approach to cleavage site determination
Source: PLoS One. 2017 Jun 27;12(6):e0179853. doi: 10.1371/journal.pone.0179853 (PMC5487053; doi:10.1371/journal.pone.0179853)
Supplement: S2 File — (PDF) [file pone.0179853.s002.pdf]

Guide to differences between ER2566 and BL21(DE3)

Brian Anton, William Jack, James Samuelson, Deborah Dila, Julie Menin and Elisabeth A. Raleigh

Supplement to: EcoBLMcrX, a Classical Modification-Dependent Restriction Enzyme in *Escherichia coli* B: Characterization *in vivo* and *in vitro* with a New Approach to Cleavage Site Determination.

Alexey Fomenkov, Zhiyi Sun, Deborah Dila, Brian Anton, Richard J. Roberts, Elisabeth A. Raleigh.

Abstract

ER2566 (NEB T7 Express) was developed as an alternative to BL21(DE3) better suited to expression and purification of DNA-active enzymes, such as restriction enzymes, methyltransferases, exonucleases, polymerases and binding proteins. The strain background is the same, BL21, taking advantage of the good expression and growth properties of the B line. The response to DNA damage, expression of nonspecific nuclease and modification-dependent enzymes and T1-phage resistance have been altered by introduction of suitable alleles from K-12, resulting in about 6% K-12 content in a BL21 background. The λDE3 prophage is absent, with T7 *gene1* placed into *lacZ*.

Introduction

The BL21(DE3) T7 expression system: Overexpression of potentially toxic proteins is at present best achieved by the use of a system based upon the bacteriophage T7 RNA polymerase (T7 *gene1*). This system as improved has three advantages: first, extremely low expression from the overexpression vector in the absence of T7 *gene1*; second, very high gene expression upon induction of expression of T7 *gene1* in suitable

bacterial hosts, due to the use of transcription and translation from signals originally found in phage T7; and the standard use of a protease-deficient host strain that has good growth properties. Good growth behavior is characteristic of *E. coli* B. BL21(DE3), a derivative of *E. coli* B, is naturally deficient in the protease Lon, and this strain also lacks OmpT.

Problems with the system, and summary of fixes. Five difficulties remain with the T7 expression system. To address these, six patches of K-12 DNA (~264 kb) were introduced into BL21, the parent of the lysogen BL21(DE3), in 11 transductional steps. DNA of K-12 origin accounts for about 6% of the ER2566 sequence. 222 K-12 genes have replaced 293 BL21(DE3) genes in these recombinant patches. In addition, one engineered mutation introduced the T7 *gene1* into the *lacZ* gene, and a transposon insertion inactivated a modification-dependent endonuclease.

In descending order of generality, the problems addressed were:

DNA damage induces the prophage carrying T7 *gene1*. Due to the presence of the viable prophage λDE3 that carries *gene1*, premature lysis of the strain can be caused by expression of even low levels of proteins that lead to DNA damage and consequent induction of the SOS regulon. BL21 was engineered to place T7 gene1 into the *lac* operon under *lacI* control. A 12 kb cryptic lambdoid prophage without lysis functions, λ*B, is endogenous to the *E. coli* B lineage including BL21. It remains at the λ attachment site.

Defective recovery from SOS induction. Cells can tolerate a substantial amount of chronic DNA damage as long as repair is allowed to proceed, but if they are unable to divide following repair they will be hypersensitive to any DNA damage that may occur.

This is the situation with BL21(DE3). The division defect is suppressed with a sulA promoter mutation introduced from K-12.

Susceptibility to T1-like phages. BL21(DE3) is sensitive to infection by the virulent, desiccation-resistant bacteriophage T1 and similar phages--that is, it makes the cell-surface receptor, FhuA, that these phage use for adsorption. An *fhuA* mutation introduced from K-12 prevents T1 infection.

Endonuclease I reduces quality of plasmid and DNA enzyme preparations. Endonuclease I is expressed by BL21(DE3). This non-sequence-specific DNA-degrading enzyme is found in the periplasm. Its action partially degrades plasmid preparations made from the strain and interferes with study of DNA-active enzymes. A mutation in *endA* was introduced from K-12. The construction strategy resulted in inversion of a segment of DNA near *endA*, and introduction of two IS10 elements.

Modification-dependent endonucleases (MDE). BL21(DE3) expresses three modification-dependent endonucleases, which degrade DNA when modified bases are present in particular sequence contexts: McrBC (RglB) and EcoBLMcrX (formerly $RglA_B$) and Mrr. A deletion of the restriction cluster, *Δ(yjiT-opgB)114*, was introduced from K-12. The deletion removes *mcrBC* and *mrr*, as well as remaining portions of the Type I restriction system not removed by the *hsdS* mutation of BL21(DE3). A transposon insertion upstream of *ecoBLmcrX* inactivates its function.

Other *E. coli* B functions missing in ER2566. Because the wild isolates B and K-12 differ in numerous genome islands, two B-specific genome islands were lost during construction. One 28-gene island, Phev*B, includes 8 that are putatively related to Type

II secretion. An 11-gene island adjacent to *tsr* is likely to specify degradation of 4-hydroxyphenylacetate in B as it does in *E. coli* W [1].

I. Phenotypic features of ER2566 (T7 Express) that differ from BL21(DE3)

A. No SOS-inducible prophage is present (no λ(DE3)), and T7 RNA polymerase (*gene1*) is in a different location

Why does that matter? Even minor expression of a DNA damaging protein can cause induce the DNA damage response (SOS). SOS induction of λ leads to expression of lethal prophage genes including *kil* and lysis genes R, Rz and S.

How does SOS lead to prophage expression? I thought DE3 couldn't excise? The DE3 prophage is normally dormant in the host chromosome, expressing only the phage repressor (cI^{imm21}), which represses all other phage-specific functions [2], and the Lac repressor that represses *gene1* expression [3]. An operon consisting of *lacI, lacZp* and T7 *gene1* interrupts the integrase gene of prophage λ in DE3, reducing excision capacity. In the presence of DNA damage, the coprotease activity of RecA is stimulated by products of DNA damage [4] to degrade LexA [5-8] and many phage repressors including cI^{imm21}. The SOS-inducing signal is thought to consist of single-stranded regions of DNA [9]. When the phage repressor is degraded, other phage-specific functions are expressed, including Kil [10] and lysis proteins R, Rz and S. The defect in the integrase (Int) [3] should prevent production phage particles unless an aberrant excision results in a viable particle. Still, induction, replication and expression of such phages is not prevented [11-13]. The prophage does not contain mutations (N-O- or N-P-) that would allow the cell to survive the induced state [14].

Is the lambda attachment site empty in ER2566? No, the ancestral 12 kb prophage-like element BL21, λ*B is present, as in all tested derivatives of *E. coli* B (including ER2566) except those where DE3 has replaced it such as BL21(DE3) [15]. ancestor of both ER2566 and BL21(DE3), does not carry the DE3 prophage. This element has no genes for lysis or structural proteins. The λ*B integrase genes (~1.8 kb) are homologous to lambda, but the rest is unrelated. Some genes may have terminase and tail assembly functions, according to NCBI [16]. There is no obvious repressor to be induced, and no suggestion of SOS-sensitivity.

Where is the T7 gene 1 in ER2566? We eliminated SOS-mediated λ induction by placing into the chromosomal *lacZ* gene a DNA fragment carrying T7 *gene1* (Figure A), instead of in a prophage.

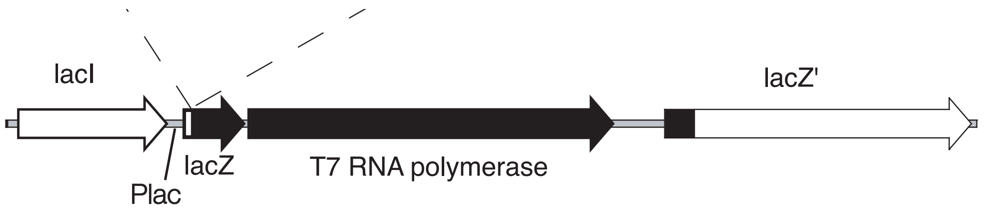

Figure A. Organization of the *lac* operon with T7 *gene1*. A short N-terminal fragment of LacZ with α-complementing activity is expressed along with the T7 RNA polymerase when *lacZp* is induced.

Expression is directed by *lacp* and regulated by the native *lacI* gene encoding the Lac repressor, eliminating the need for the DE3 prophage. The *lacZ* gene is disrupted at 440 nt from the start codon; 396 nt are deleted, 40 nt added (including 26 nt of the T7 sequence immediately upstream of *gene1*), followed by *gene1* in frame 1. A 150 aa LacZ

fragment is predicted from the sequence; this fragment restores β-galactosidase activity to when F' *Δ(lacZ)M15* is present (LacZ α-complementation).

<u>How this was done</u>

1. Overview: Placement of T7 *gene1* into the lac operon.

The T7 *gene1* was placed in the context of the *lac* operon by a series of plasmid constructions outlined in Figure B. The T7 *gene1* was cloned into the context of *lacZ* excised with XbaI, circularized without a replication origin, electroporated into BL21, and KanR transformants were selected. An ampicillin sensitive, plasmid-free strain expressing T7 RNA polymerase was chosen to carry further. The duplicated segment from the integration was eliminated by selection for loss of the sucrose-resistance and screening for loss of drug resistance, retention of T7 RNA polymerase activity and lack on LacZ activity. One strain was designated WJ56 (see S1 Figure and S1 Table).

2. Details:

Sources: All enzymes were from NEB and used in accordance with the recommendations of the supplier. *Escherichia coli* strain BL21 and T7 phage 4107 were provided by F.W. Studier (Brookhaven).

Media: Selections and screens used LB+5% sucrose+40 μg/ml X-gal; LB kanamycin (50 μg/ml), and LB ampicillin (100 μg/ml).

Plasmid constructions (all carry *bla* and are AmpR):

pCDU2 places T7 *gene1* in the context of *lacZ*. pAR1219 is a pBR322 derivative that contains T7 *gene1* (the T7 RNA polymerase gene) downstream of the *lacI* gene and the *lacUV5* promoter [17]. A 2.7 kb ClaI/BstBI fragment carrying *lac* was isolated from pRS415 [18] and ligated to the pAR1219. The added fragment contains carboxy terminal

sequences of *lacZ* and amino terminal sequences of *lacY*. An isolate with the *lac* CDS in the same orientation as the *lacI* and T7 *gene1* CDSs was named pCDU2.

pATC1: Linkers were used to add an XbaI site pCDU2 at an Ecl136I site (MBI Fermentas), to yield pATC1.

pATD2: To add selectable (*npt*) and counter-selectable (*sacB*) markers to the plasmid, a 3.8 kb SalI fragment from pBIP3 [19] was added at the SalI site of pATC1, and screened for orientation such that *npt* lay adjacent to the *lacI* sequences (Figure B).

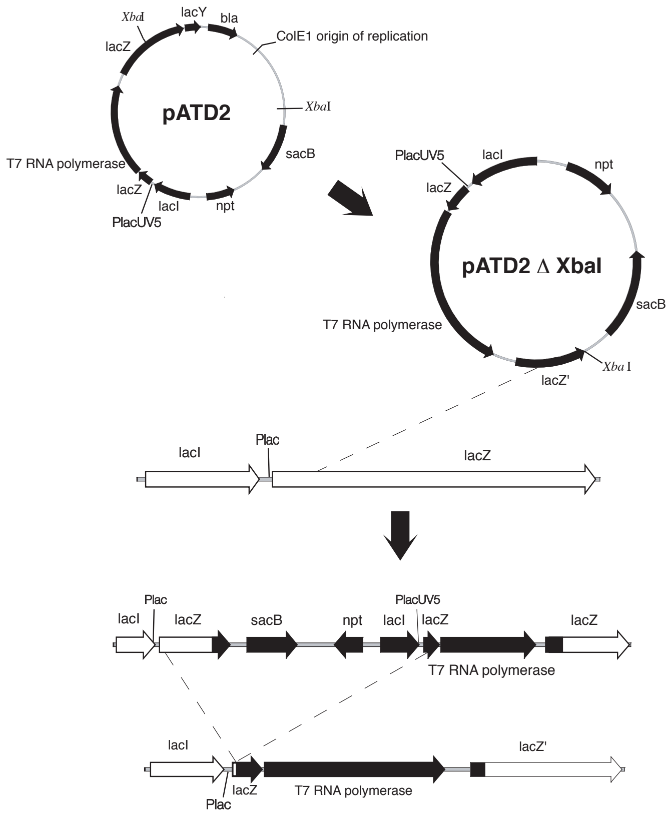

Figure B. Construction of *lacZ::T7 gene1*. Integration construct: pATD2ΔXba in Figure B is a ~10 kb nonreplicating XbaI fragment used for chromosomal integration of the constructed sequence. The isolated fragment was ligated at 1 μg/ml DNA to favor

circularization, electroporated into BL21 cells, and plated onto kanamycin plates. Transformants were screened to eliminate background vector contamination (AmpS, no plasmid DNA in miniprep [20]). Transformants that were KanR AmpS plasmidless were screened for expression of T7 RNAP using 4107. This T7 variant lacks RNAP activity, thus requiring RNAP production by the host to make normal plaques. One strain meeting this criterion was used for subsequent work.

Resolution of integrant: A single crossover integration results in duplication of regions of the *lacI* and *lacZ* gene regions, flanking *sacB-npt* (second linear schematic in Figure B). Expression of *sacB* is toxic [19]. Sucrose selection was used to select for segregants that excised the *sacB-npt* segment. LB sucrose Xgal lacking salts or drugs. ~50% of the colonies were blue, as expected if the resolution step reversed the insertion event. Individual white colonies were retested separately for Lac and Kan phenotypes. ~10% of the colonies were KanR, and these were not further analyzed. The remaining 10 strains were tested for their ability to support the growth of phage 4107. Two were able to complement the T7 RNA polymerase deletion and were Lac^- (white on X-gal plates). This was named WJ56.

B. Improved recovery from SOS-induction

Transductions from K-12 introduced the *sulA211* allele, a promoter mutation, along with surrounding sequence. Reduced *sulA* expression allows improved recovery from DNA damage in the presence of the endogenous *lon* defect of many B strains.

Cells can tolerate a substantial amount of chronic DNA damage if the damage is repaired [21]. However, if they are unable to divide following repair, they will be hypersensitive SOS induction, even without an inducible prophage. The SulA (SfiA) and

Lon products are induced by SOS. The SulA protein inhibits cell division during the DNA repair process, so that only cells with intact chromosomes attempt to divide [22]. One function of the Lon protease is to degrade SulA [23]. SulA levels are maintained by continued synthesis until repair is complete and LexA repression is reimposed. If SulA is not degraded, the cells undergo lethal filamentation. BL21(DE3) and ER2566 are Lon-deficient like other B strains [15, 24]. BL21, the ancestor of both strains, is sensitive to DNA damage caused by ultraviolet light, but ER2566 is much less so, due to the promoter mutation *sulA211*.

C. Phage T1 and relatives don't infect

ER2566 carries an IS2 insertion in *fhuA*, introduced from a K-12 strain by transduction. This codes for a receptor used by virulent, desiccation-resistant bacteriophage such as T1 to adsorb to the cell during infection [25]. BL21(DE3) makes this receptor and is T1-sensitive. Such phages are a recurrent problem in fermentation facilities because they are airborne, virulent, fast-growing and resistant to many inactivation procedures. Note that temperate phage phi80 also uses this receptor.

D. Nonspecific Endonuclease I is absent

The mutation *endA1-E208K*was introduced from K-12 using a genetically-linked Tn10 insertion, with subsequent selection for tetracycline sensitivity [26]. This periplasmic non-sequence-specific DNA-degrading enzyme. It is sometimes difficult to eliminate this nuclease from preparations of nucleases and DNA-modifying enzymes with more useful properties. Plasmid preparations from EndA$^-$ are also cleaner, with fewer nicks and breaks. Mutations of the *endA* gene eliminate the enzymatic activity [27], which is nonessential for cell growth and health under laboratory conditions.

E. ER2566 does not restrict DNA with ^{m6}A, ^{m5}C or ^{hm5}C

What genes restrict modified DNA in BL21(DE3)? Three modification-dependent restriction enzymes (MDE) of BL21(DE3) are McrBC (RglB), Mrr and EcoBLMcrX (formerly McrA, RglA). These prevent maintenance of some site-specific modification methyltransferases (MTases). Each MDE restricts entering DNA modified in particular sequence contexts [28]. An MTase required to protect against cleavage by a cognate restriction endonuclease (RE) may create an MDE-sensitive site, so that neither the RE nor the MTase can be expressed.

How were these inactivated? E. coli K-12 and B each carry a cluster of homologous restriction genes including *mcrBC, hsdRMS* (encoding the Type I restriction systems EcoKI and EcoBI) and *mrr* in a conserved genomic context. In BL21(DE3), the *hsdS* component of EcoBI has been inactivated by an IS1 insertion, but the other two systems are still present. A deletion of the whole cluster had been characterized in K-12 [29, 30]. This was introduced into ER2566 by transduction, resulting in loss of McrBC and Mrr activities as well. In addition, we isolated a new insertion inactivating EcoBLMcrX as described in the main text.

Why wasn't the K-12 mcrA deletion allele used? E. coli B is already *mcrA*-deficient because it lacks the e14 cryptic prophage that carries *mcrA* in K-12. For historical reasons, activities outside the restriction cluster in *E. coli* K-12 and B were given the same name: $mcrA_K$ ($rglA_K$) and $mcrA_B$ ($rglA_B$). However, it has long been clear that these are not homologous genes. The results in the main text demonstrate that the *E. coli* B gene is a completely new kind of restriction activity. Accordingly we are naming it *ecoBLmcrX* to clearly distinguish it from the characterized *mcrA* gene of K-12.

How this was done

Isolation of *mcr-73::Tn10dTet-tetA::IS10*, used in construction of ER2566, is described in the main text.

The contents of the BL21(DE3) restriction cluster were removed by transduction, using K-12 as the source. The *opgB::Tn10*-promoted K-12 deletion was one described earlier [29, 30], and removes K-12 DNA between *yjiT* and *opgB*, leaving behind an IS10 element. In the same transduction, an adjacent 11-gene island in BL21(DE3) was replaced with MG1655 sequence, which does not carry it. This *hpa* cluster specifies degradation of 4-hydroxyphenylacetate [1].

F. A potential type II secretion system is missing from ER2566

How did that happen? Transductional replacement of B DNA with K-12 DNA was used to bring in *endA1*. The transduction also removed a 28-gene genome island (Phev*B in [15]) of BL21(DE3) that includes 8 genes assigned to this function.

Why does it matter? It could affect secretion across the outer membrane of proteins exported via SecA and Tat-dependent pathways [31]. This system has not been specifically studied and shown to affect secretion. However, in stationary phase, minimal-medium from BL21(DE3) cultures had much more extracellular protein than the same medium from K-12 cultures, or another B strain [24].

II. Sequence overview

The following discussion makes use of alignments between the genome sequences of the two parents and the descendant (Figure C).

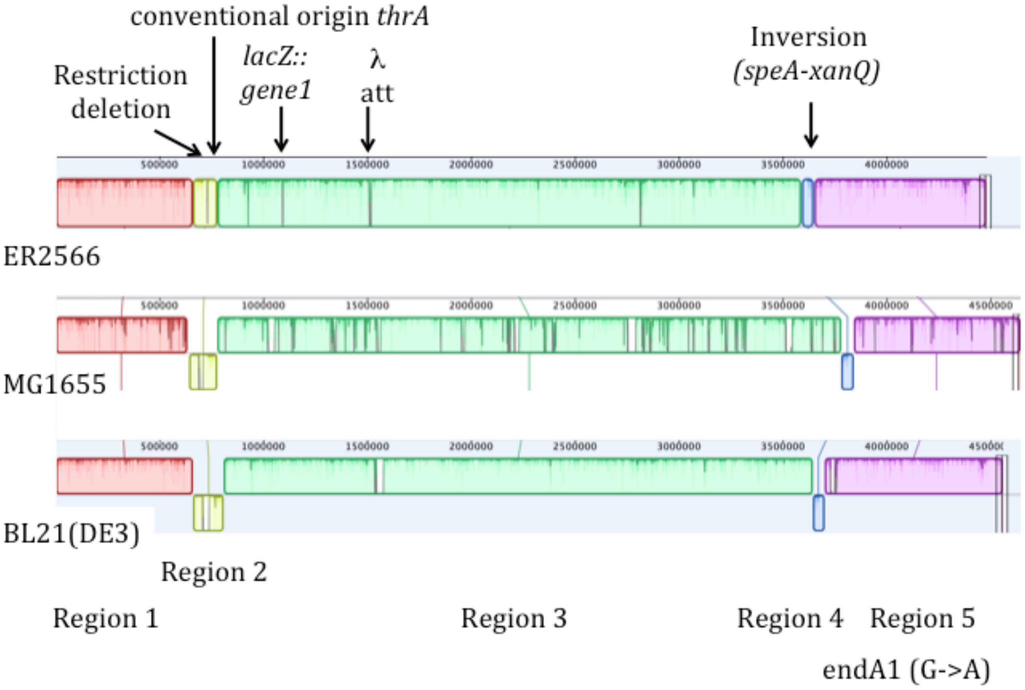

Figure C. Genome alignment of ER2566, *E. coli* K-12 str MG1655, *E. coli* B str BL21(DE3). Genetic markers discussed in the text are noted above (arrows) and below the alignment. Regions of synteny (LCB) in all three strains are the same color. Inverted regions of MG1655 and BL21(DE3) with respect to ER2566 are upside down. The conventional genetic map origin is indicated.

For clarity of presentation, the sequences start at the replication origin, rather than the conventional numerical origin. This allows visualization of the two regions inverted with respect to the parents. These inversions divide the alignment into 5 syntenic segments (called Locally Co-linear Blocks, LCB), colored and designated Regions 1-5 in Figure C. Spikes in the colors show dips in the average nucleotide identity to the consensus (majority rule). Where there are deletions or insertions in one strain relative to

the others, the spikes drop to the horizontal axis. For example at the l attachment site, BL21(DE3) has the DE3 prophage, MG1655 has nothing, and ER2566 carries the original *E. coli* B cryptic defective lambdoid prophage.

A. Two inversions relative to B and K-12 are present

One inversion was mediated by recombination between ancestral IS1 elements (see S1 Figure, Figure C). We have listed the IS1 elements that bound the inversion (numbered as reported in [24]). The transduction from K-12 that introduced the restriction deletion lies entirely within the inversion. Thus we placed the introduced K-12 deletion allele between inversion termini in the genotype: *IN(IS1_26-Δ(yjiT-opgB)114::IS10-IS1_1)*.

The other inversion is a Tn10-promoted rearrangement called a deletion/inversion selected during construction. The original Tn10 insertion was used during transduction from K-12 to introduce the linked mutation *endA1*, and was in *speA*.A TetS derivative was selected from this. The resulting arrangement has inverted DNA between that site and a site in *xanQ*, including one of the original IS10s at the insertion site. The event simultaneously deleted the unique Tn10 material including *tetRtetA*. This is a characteristic Tn10-mediated event [32-34]. To retain the signature of the original insertion we have adopted the genotype format *IN(speA::IS10-xanQ::IS10)*.

B. Six segments of K-12 DNA were introduced by transduction resulting island gain and loss.

Briefly, six patches of K-12 DNA (~264 kb) account for about 6% of the ER2566 sequence (4.5 Mb). 222 genes have replaced 293 BL21(DE3) genes in these recombinant patches. 17 variants within CDS obtained from MG1655 are private to ER2566; of these,

2 are responsible for genotypes (*fhuA2::IS2*, *endA1-E208K*). One of the private variants is in Region 2, three in Region 3 and one each in Region 4 and 5. The correlation of the genomes and the recombination patches identified are detailed in supplementary Excel files: S2 Table 3way comparison annotations LCB1-5.xlsx, S3 Table Recombination Patches and their Markers.xlsx and S4 Table Recombination Patches Short Summary.xlsx

Three *E. co*li B islands were lost during two transductions. The restriction cluster and the *hpa* genome island were replaced to eliminate restriction; the third genome island (28 genes; called Phev*B in Studier et al [15] was mentioned in section IF. It is adjacent to the *pheV* tRNA and carries genes for a proposed type II secretion system in BL21(DE3), along with genes that may have specified capsule formation but are likely inactive in BL21(DE3) [15]. The secretion system was also mentioned in reference [24].

III. Genetics Methods and Nomenclature

Genetic constructions: P1*vir* transductions, tests for auxotrophy, UV resistance, and phage resistance were by standard methods [35]. Isolation of TetS derivatives [26], random Tn10 insertions [36], and mutants sensitive to T4*gt* [37, 38] were as described. The EndA phenotype was tested by a modification of the original method [39]: chloroform was substituted for toluene, and ethidium bromide (100 μg/ml) for acridine orange. Where required, tetracycline was used at 15 μg/ml and kanamycin at 20 μg/ml. Transformations employed an RbCl method obtained from the John Innes Institute via J. Elhai.

Genetic nomenclature: Nomenclature is standard [40], with two exceptions. The first affects derivatives of *E. coli* B. The B strain naturally lacks *lon* and *dcm* [41]. Since this is the wild-type state, these deficiencies would not normally be listed in a genotype;

however, for many investigators, expectations will be based on the wild state of *E. coli* K-12, which has these functions. To avoid confusion, we have listed these in square brackets. Second, the treatment of inversions and their contents is a modification of historical practice. IN(...) signifies INversion, with the termini of the inversion within the parentheses [42]. We have included additional genotypic information between the termini

References

1. Prieto MA, Diaz E, Garcia JL. Molecular characterization of the 4-hydroxyphenylacetate catabolic pathway of *Escherichia coli* W: engineering a mobile aromatic degradative cluster. J Bacteriol. 1996;178(1):111-20. PubMed PMID: 8550403.

2. Roberts JW, Devoret R. Lysogenic induction. In: Hendrix R, Roberts J, Stahl F, Weisberg R, editors. Lambda II. Cold Spring Harbor: Cold Spring Harbor Laboratory; 1983. p. 123-44.

3. William Studier F, Rosenberg AH, Dunn JJ, Dubendorff JW. [6] Use of T7 RNA polymerase to direct expression of cloned genes. Meth Enzymol. 1990;185:60-89. doi: 10.1016/0076-6879(90)85008-c.

4. Little JW. Mechanism of specific LexA cleavage: autodigestion and the role of RecA coprotease. Biochimie. 1991;73(4):411-21.

5. Craig NL, Roberts JW. Function of nucleoside triphosphate and polynucleotide in *Escherichia coli* recA protein-directed cleavage of phage lambda repressor. J Biol Chem. 1981;256(15):8039-44.

6. Little JW, Edmiston SH, Pacelli LZ, Mount DW. Cleavage of the *Escherichia coli* lexA protein by the recA protease. Proc Natl Acad Sci USA. 1980;77(6):3225-9.

7. Witkin EM. The radiation sensitivity of *Escherichia coli* B: a hypothesis relating filament formation and prophage induction. Proc Natl Acad Sci USA. 1967;57:1275-9.

8. Witkin EM. RecA protein in the SOS response: milestones and mysteries. Biochimie. 1991;73(2-3):133-41.

9. Sassanfar M, Roberts JW. Nature of the SOS-inducing signal in *Escherichia coli*: the involvement of DNA replication. J Mol Biol. 1990;212:79-96.

10. Sergueev K, Yu D, Austin S, Court D. Cell toxicity caused by products of the p(L) operon of bacteriophage lambda. Gene. 2001;272(1-2):227-35. PubMed PMID: 11470529.

11. Herskowitz I, Signer ER. A site essential for expression of all late genes in bacteriophage l. J Mol Biol. 1970;47:545-56.

12. Stevens WF, Adhya S, Szybalski W. Origin and bidirectional orientation of DNA replication in coliphage lambda. In: Hershey AD, editor. The Bacteriophage Lambda. Cold Spring Harbor: Cold Spring Harbor Laboratory; 1971. p. 515-34.

13. Imae Y, Fukasawa T. Regional replication of the bacterial chromosome induced by derepression of prophage lambda. J Mol Biol. 1970;54(3):585-97. doi: http://dx.doi.org/10.1016/0022-2836(70)90129-4.

14. Calef E, Avitabile A, del Giudice L, Marchelli C, Menna T, Neubauer Z, et al. The genetics of the anti-immune phenotype of defective lambda lysogens. In: Hershey AD, editor. The Bacteriophage Lambda. Cold Spring Harbor, N.Y.: Cold Spring Harbor Laboratory; 1971. p. 609-20.

15. Studier F, Daegelen P, Lenski R, Maslov S, Kim J. Understanding the Differences between Genome Sequences of *Escherichia coli* B Strains REL606 and BL21(DE3) and

Comparison of the *E. coli* B and K-12 Genomes. J Mol Biol. 2009. doi: 10.1016/j.jmb.2009.09.021. PubMed PMID: 19765592.

16. Marchler-Bauer A, Zheng C, Chitsaz F, Derbyshire MK, Geer LY, Geer RC, et al. CDD: conserved domains and protein three-dimensional structure. Nucleic Acids Res. 2012. doi: 10.1093/nar/gks1243. PubMed PMID: 23197659.

17. Davanloo P, Rosenberg AH, Dunn JJ, Studier FW. Cloning and expression of the gene for bacteriophage T7 RNA polymerase. Proceedings of the National Academy of Sciences of the United States of America. 1984;81(7):2035-9. PubMed PMID: 6371808; PubMed Central PMCID: PMCPMC345431.

18. Simons RW, Houman F, Kleckner N. Improved single and multicopy *lac*-based cloning vectors for protein and operon fusions. Gene. 1987;53(1):85-96. PubMed PMID: 3596251.

19. Slater S, Maurer R. Simple phagemid-based system for generating allele replacements in *Escherichia coli*. J Bacteriol. 1993;175:4260-2.

20. Sambrook J, Fritsch EF, Maniatis T. Molecular Cloning: A Laboratory Manual. 2nd ed. New York: Cold Spring Harbor Press; 1989.

21. Heitman J, Zinder ND, Model P. Repair of the *Escherichia coli* chromosome after *in vivo* scission by the *Eco*RI endonuclease. Proc Natl Acad Sci USA. 1989;86:2281-5.

22. Huisman O, D'Ari R, Gottesman S. Cell-division control in *Escherichia coli*: specific induction of the SOS function SfiA protein is sufficient to block septation. Proc Natl Acad Sci USA. 1984;81:4490-4.

23. Mizusawa S, Gottesman S. Protein degradation in *Escherichia coli:* the *lon* gene controls the stability of sulA protein. Proc Natl Acad Sci USA. 1983;80:358-62.

24. Jeong H, Barbe V, Lee CH, Vallenet D, Yu DS, Choi SH, et al. Genome sequences of Escherichia coli B strains REL606 and BL21(DE3). J Mol Biol. 2009;394(4):644-52. doi: 10.1016/j.jmb.2009.09.052. PubMed PMID: 19786035.

25. Bachmann B. Derivations and genotypes of some mutant derivatives of *Escherichia coli* K-12. In: Neidhardt FC, Ingraham JL, Low KB, Magasanik B, Schaechter M, Umbarger HE, editors. *Escherichia coli* and *Salmonella typhimurium*: cellular and molecular biology. 2. Washington, D.C.: American Society for Microbiology; 1987. p. 1190-219.

26. Bochner BR, Huang HC, Schieven GL, Ames BN. Positive selection for loss of tetracycline resistance. J Bacteriol. 1980;143(2):926-33. PubMed PMID: 6259126; PubMed Central PMCID: PMCPMC294396.

27. Dürwald H, Hoffmann-Berling H. Endonuclease-I-deficient and ribonuclease I-deficient Escherichia coli mutants. J Mol Biol. 1968;34(2):331. PubMed PMID: 9130631438174094787related:w4Vht-qEtn4J.

28. Loenen WA, Raleigh EA. The other face of restriction: modification-dependent enzymes. Nucleic Acids Res. 2014;42(1):56-69. doi: 10.1093/nar/gkt747. PubMed PMID: 23990325; PubMed Central PMCID: PMCPMC3874153.

29. Raleigh EA, Trimarchi R, Revel H. Genetic and physical mapping of the mcrA (rglA) and mcrB (rglB) loci of Escherichia coli K-12. Genetics. 1989;122(2):279-96. Epub 1989/06/01. PubMed PMID: 2548920; PubMed Central PMCID: PMCPMC1203701.

30. Kelleher JE, Raleigh EA. A novel activity in *Escherichia coli* K-12 that directs restriction of DNA modified at CG dinucleotides. J Bacteriol. 1991;173(16):5220-3.

Epub 1991/08/01. PubMed PMID: 1830580; PubMed Central PMCID: PMCPMC208216.

31. Korotkov KV, Sandkvist M, Hol WGJ. The type II secretion system: biogenesis, molecular architecture and mechanism. Nat Rev Microbiol. 2012;10(5):336-51. doi: 10.1038/nrmicro2762. PubMed PMID: 22466878; PubMed Central PMCID: PMCPMC3705712.

32. Kleckner N, Reichardt K, Botstein D. Inversions and deletions of the *Salmonella* chromosome generated by the translocatable tetracycline resistance element Tn10. J Mol Biol. 1979;127:89-115.

33. Raleigh EA, Kleckner N. Multiple IS10 rearrangements in *Escherichia coli*. J Mol Biol. 1984;173(4):437-61. Epub 1984/03/15. doi: 0022-2836(84)90390-5 [pii]. PubMed PMID: 6323719.

34. Shen MM, Raleigh EA, Kleckner N. Physical analysis of Tn10- and IS10-promoted transpositions and rearrangements. Genetics. 1987;116(3):359-69. Epub 1987/07/01. PubMed PMID: 3038673; PubMed Central PMCID: PMC1203147.

35. Sternberg NL, Maurer R. Bacteriophage-mediated generalized transduction in Escherichia coli and Salmonella typhimurium. Methods Enzymol. 1991;204:18-43. doi: http://dx.doi.org/10.1016/0076-6879(91)04004-8. PubMed PMID: 1943777.

36. Kleckner N, Bender J, Gottesman S. Uses of transposons with emphasis on Tn10. Methods Enzymol. 1991;204:139-80. doi: 10.1016/0076-6879(91)04009-D. PubMed PMID: 1658561.

37. Georgopoulos CP. Isolation and preliminary characterization of T4 mutants with nonglucosylated DNA. Biochem Biophys Res Commun. 1967;28(2):179-84. PubMed PMID: 5340730.

38. Revel HR. Restriction of nonglucosylated T-even bacteriophage: properties of permissive mutants of Escherichia coli B and K12. Virology. 1967;31(4):688-701. Epub 1967/04/01. PubMed PMID: 4290282.

39. Zabel DJ, Trucksis M, Depew RE. *Salmonella typhimurium* mutants with reduced levels of transfer ribonucleic acid-inhibitable endodeoxyribonucleolytic activity. J Bacteriol. 1980;144:173-8.

40. Demerec M, Adelberg EA, Clark AJ, Hartman PE. A proposal for a uniform nomenclature in bacterial genetics. Genetics. 1966;54:61-76.

41. Daegelen P, Studier F, Lenski R, Cure S, Kim J. Tracing Ancestors and Relatives of Escherichia coli B, and the Derivation of B Strains REL606 and BL21(DE3). J Mol Biol. 2009. doi: 10.1016/j.jmb.2009.09.022. PubMed PMID: 19765591.

42. Hill CW, Harnish BW. Inversions between ribosomal RNA genes of *Escherichia coli*. Proc Natl Acad Sci USA. 1981;78:7069-72.
