# Supplementary material for: EcoBLMcrX, a classical modification-dependent restriction enzyme in Escherichia coli B: Characterization in vivo and in vitro with a new approach to cleavage site determination
Source: PLoS One. 2017 Jun 27;12(6):e0179853. doi: 10.1371/journal.pone.0179853 (PMC5487053; doi:10.1371/journal.pone.0179853)
Supplement: S3 File — (PDF) [file pone.0179853.s003.pdf]

Complete WebLogo Results--MDE digests of variably modified substrates

Zhiyi Sun and Elisabeth A. Raleigh

Supplement to: EcoBLMcrX, a Classical Modification-Dependent Restriction Enzyme in *Escherichia coli* B: Characterization *in vivo* and *in vitro* with a New Approach to Cleavage Site Determination.

Alexey Fomenkov, Zhiyi Sun, Deborah Dila, Brian Anton, Richard J. Roberts, Elisabeth A. Raleigh.

Methodology

The program WebLogo 3 [1, 2] was employed to identify and visualize over-represented motif sequences at the sites of cleavage--that is, the sites at which the adaptors had ligated most often.

For this analysis, the amount of data was too large to compute in its entirety. Instead, we considered only favored cleavage sites. The number of reads (X) that align to a particular genomic location was defined as digestion frequency. The top N favored loci were considered with WebLogo. N was derived by considering the number of potential sites (Y) in the substrate and the total number of reads (Z). If the reads were randomly distributed, they would end at the site with a frequency of Z/Y. A locus is considered high-frequency (and included in N) if X> Z/Y.

Sequence logos represent the information content at a particular position – how conserved or how highly specified it is. The overall height of each stack is proportional to the sequence conservation, defined as the difference between the maximum possible entropy and the entropy of the observed symbol distribution, at that position. The maximum sequence conservation per site is 2 for DNA and RNA ($\log_2 4$) when all the

alignments contain a specific base at a given position. If each of the four bases is equally represented at that position, the height is zero bits. The height of each symbol (A,T, G,C for DNA) is proportional to the observed frequency of the corresponding nucleotide. If two bases are equally represented, the sequence conversation (overall stack height) is 1 bits, and both bases appear as 0.5 bits in the logo. When generating the sequence logo, the frequencies of digestion events at a particular base sequence context (normalized to the sequence occurrence in the reference sequence, Y/Z) also contribute to the strength of the signal (i.e., the height of the stack).

Coordinates in this discussion refer to positions in a 5-base sequence, with the convention of Figure 1 of the main text in parentheses. Figure A and logo figures adhere to the convention of Figure 1 of the main text.

Results and Discussion

(1) When C is methylated in all contexts, as in XP12, the restriction sequence of all the 3 enzymes is [AG]CN[AG]C—the 1st (-2) and 4th (+2) base can be either G or A.

(2) When C is only methylated in the GC context—e.g., pBR322.M.CviPI (GmC) and pBR322*fnu4HIM* (GmCNGC)--the 1st (-2) position is always G for all the 3 enzymes suggesting the cytosine at position 2 (-1) is required to be methylated.

(3) When C is only methylated in CpG context—e.g., pBR322.M.SssI (mCpG)--the preferred restriction sequence is GmCG[AG]mCG for NhoI and GC[GC][AG]mCG for

EcoBLMcrX, suggesting that both NhoI and EcoBLMcrX favor methylated cytosine at position 5 (+3) (GmCNGmC) over the unmodified base.

Interestingly, it appeared that for EcoBLMcrX the requirement of modified cytosine at position 2 (-1) was greatly relaxed when the cytosine at 5 (+3) is methylated and meanwhile a methylated cytosine at the -1 (-3) position seems to be favored (i.e., mCGC[GC][AG]mCG). The over-representation of a methylated cytosine at -1 (-3) is also observed for NhoI (mC GmCG[AG]mCG). These results imply that loci with multiple methylated cytosines are preferred substrate for both NhoI and EcoBLMcrX. In comparison, BceYI does not show a strong preference for substrates with multiple methylated cytosines.

The fact that both NhoI and EcoBLMcrX digest pBR322*fnu4HIM* (GmCNGC), which contains only one methylated cytosine on one strand but two methylated cytosines on double strands, suggests the requirement of multiple modified cytosines is not limited to one strand—as long as there are multiple modified cytosines from the two strands combined, these two enzymes would recognize and cut. Overlapping recognition sites can also result in an additional modification of the C in position 5 (+3) (see Figure A):

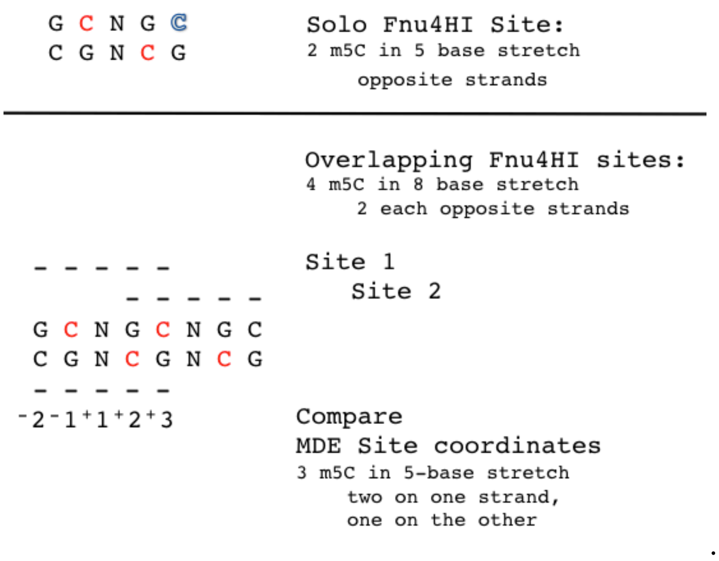

Figure A. Obtaining dense modification with M.Fnu4HI--overlapping sites

It appears that both NhoI and EcoBLMcrX cut the GmC substrate (pBR322.M.CviPI) more efficiently than the GmCNGC substrate (pBR322*fnu4HIM*). The GmC substrate has four methylated cytosines in the GCNGC context (both strands combined) whereas the GmCNGC substrate has only 2 modified bases. Again this implies a positive correlation between NhoI and EcoBLMcrX's activity and the methylation level of the substrate.

(4) All 3 enzymes seem to have more relaxed recognition on hydroxymethylated substrates (or more star activities). This could reflect the ability to accept the methyl group of T in a low GC environment

(5) The observed enrichment of G/C at the 3rd (+1) base is probably due to ligation bias because when the single-base overhang is G or C, they pair more efficiently than the A-T pair.

S3 File WebLogo Results

NOTE The sequence logos below were generated from all the restriction events at high-frequency digestion loci as described above, so that the sequences of preferred loci got more weight in logo generation.

Control Fnu4HI

pUC19

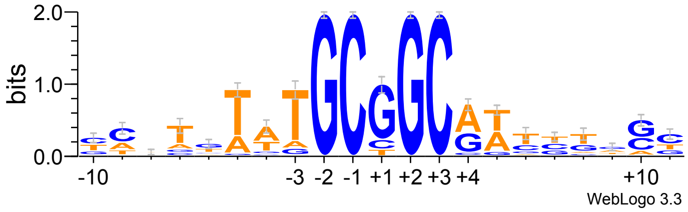

S3 File WebLogo Results

BceYI

XP12 (all cytosines methylated)

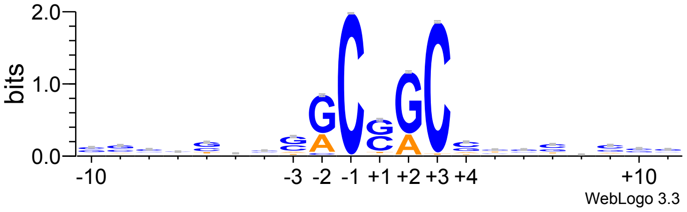

pBR322*fnu4HIM* (GmCNGC)

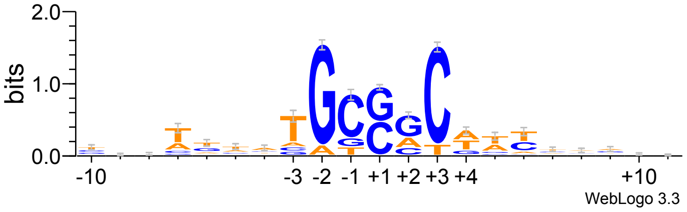

pBR322-MCviPI (GmC methylation)

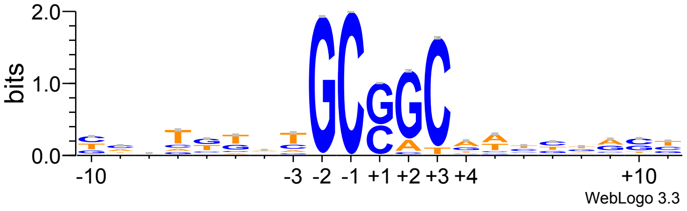

pBR322-MSssI (mCpG methylation)

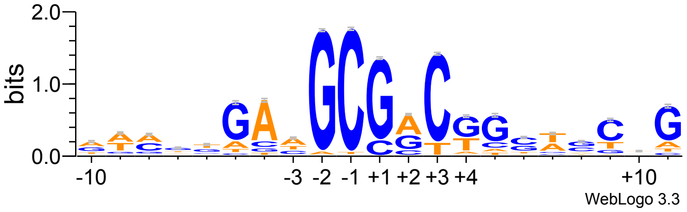

T4 (all cytosine hydroxymethylated)

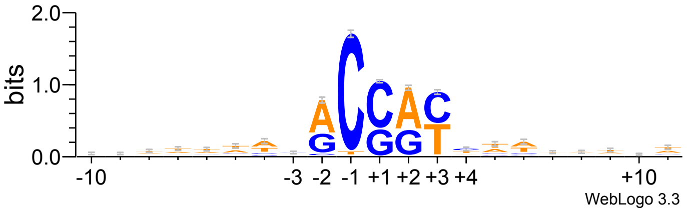

NhoI

XP12 (all cytosines methylated)

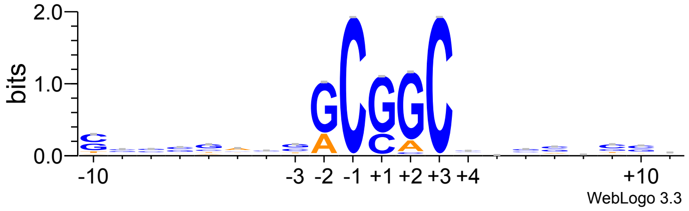

pBR322*fnu4HIM* (GmCNGC)

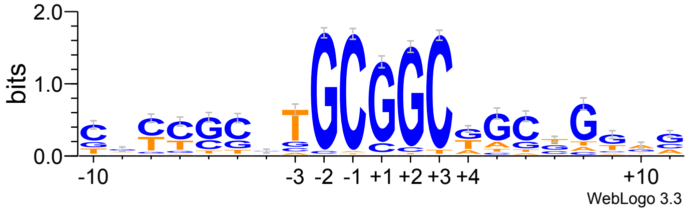

pBR322-MCviPI (GmC methylation)

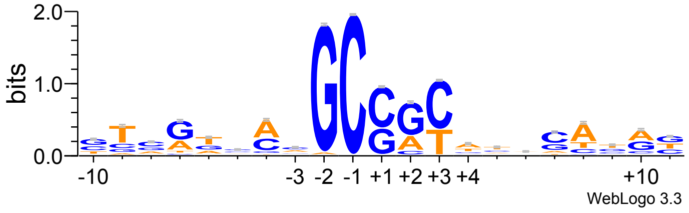

pBR322-MSssI (mCpG methylation)

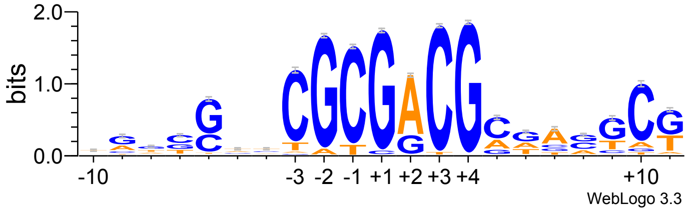

T4 (all cytosine hydroxymethylated)

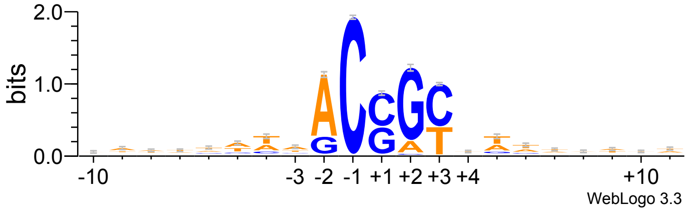

EcoBLMcrX

XP12 (all cytosines methylated)

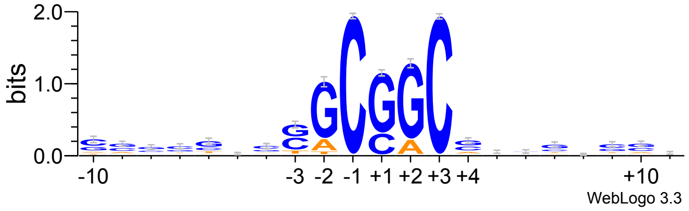

pBR322*fnu4HIM* (GmCNGC)

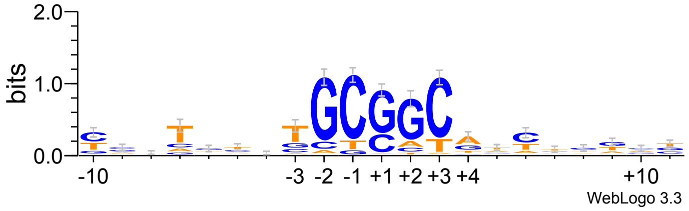

pBR322*bbVIM* (GmCAGC)

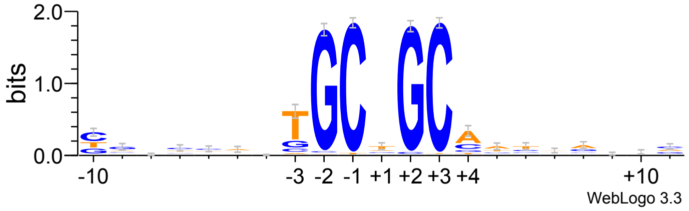

pBR322-MCviPI (GmC methylation)

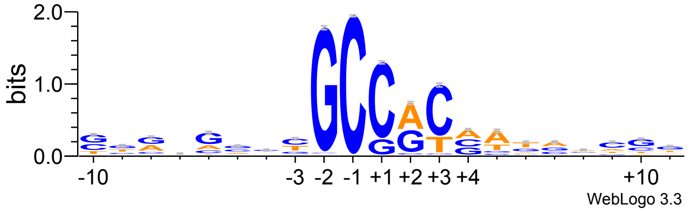

pBR322-MSssI (mCpG methylation)

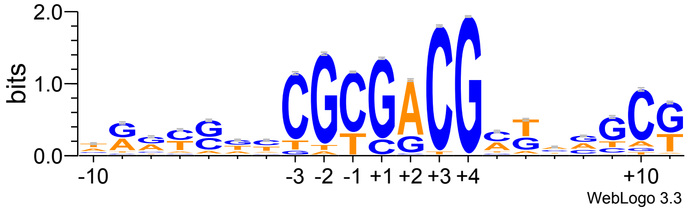

T4 (all cytosine hydroxymethylated)

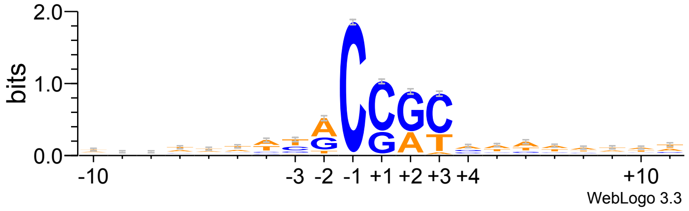

1. Crooks GE, Hon G, Chandonia JM, Brenner SE. WebLogo: a sequence logo generator. Genome Res. 2004;14(6):1188-90. doi: 10.1101/gr.849004. PubMed PMID: 15173120; PubMed Central PMCID: PMCPMC419797.

2. Schneider TD, Stephens RM. Sequence logos: a new way to display consensus sequences. Nucleic Acids Res. 1990;18(20):6097-100. PubMed PMID: 2172928; PubMed Central PMCID: PMCPMC332411.
