# Supplementary material for: EcoBLMcrX, a classical modification-dependent restriction enzyme in Escherichia coli B: Characterization in vivo and in vitro with a new approach to cleavage site determination
Source: PLoS One. 2017 Jun 27;12(6):e0179853. doi: 10.1371/journal.pone.0179853 (PMC5487053; doi:10.1371/journal.pone.0179853)
Supplement: S1 Table — (PDF) [file pone.0179853.s008.pdf]

S1 Table

Supplement to:

EcoBLMcrX, a Classical Modification-Dependent Restriction Enzyme in *Escherichia coli* B: Characterization *in vivo* and *in vitro* with a New Approach to Approach to Cleavage Site Determination. Alexey Fomenkov, Zhiyi Sun, Deborah K. Dila, Brian P. Anton, Richard J. Roberts, Elisabeth A. Raleigh.

S1 Table Part A ER2566 Construction Steps

Strain	Genotype*	Construction/Source	Relevant sequence block	S3 table segment detail	Notes
BL21	*hsdS gal ompT [lon] [dcm]*	F.W. Studier			has lambda*B
ER2374	*hsdS gal ompT [lon] [dcm] IN(IS1_26-IS1_1)*	Colony isolation	=LCB2; Unselected inversion		116 kb spontaneous inversion
ER2383	*BL21 (hsdS-B or hsdR2-K) mcrB1? zjj202::Tn10 serB28*	BL21(=ER2374) X P1(ER1384) -->TetR (Ser-)	in LCB2		*opgB202::Tn10*
ER2384	ER2383 *Δ(opgB-yjiT)114::IS10* Ser+	ER2383 X P1(ER1700) --> Ser+ (TetS McrB-)	In LCB2	LCB2no1 recomb patch	
ER2451	ER2384 *sulA::Tn5 pyrD*	ER2384 X P1(ER1579)--> KanR (Ura- UVR).	In LCB3	LCB3no5 recomb patch	
ER2457	ER2451 *sulA11* Pyr+	ER2451 X P1(ER1566)--> Ura+ (KanS)	In LCB3	LCB3no5 recomb patch	*sfiA11* now known as *sulA211* (CGSC)
ER2458	ER2457 *pan-6 zad::Tn10 fhuA2*	ER2457 X P1(ER2391)--> TetR (Pan-, f80R).	In LCB3	LCB3no1 recomb patch	*fhuB478::Tn10* ? this is in RK4375
ER2467	ER2458 Pan+ TetS	ER2458 X P1(1700)--> Pan+(TetS)	In LCB3	LCB3no1 recomb patch	

S1 Table

ER2479	ER2467 *mcr-73::(Tn10* dTet (TetR))	ER2467 X P1(ER2475)--> TetR (T4gtS)	In LCB3	LCB3no4 K-B island	mutagenesis
ER2488	ER2479 *mcr-73::(Tn10* dTet *-tetA::IS10)2*	ER2479 -->TetS	In LCB3	LCB3no4 K-B island	TetS selection
ER2522	ER2488 *speA210::Tn10 endA1*	ER2488 x P1(ER2214) --> TetR (EndA-)	In LCB5	LCB4-5 edge recomb patches, LCB5 (endA1)	*speA210::Tn10*
ER2523	ER2522 *IN(speA210::IS10-xanA::IS10)2*	ER2522 --> TetS	=LCB4	LCB4	59 kb inversion
ER2554	ER2523 *proC::Tn5 zah-281::Tn10*	ER2523 x P1(ER2545)--> TetR (Pro- KanR)	In LCB3	LCB3no2 recomb patch remnant	*mhpC281::Tn10* (CGSC)
ER2566	*IN(IS1_26-IS1_1) fhuA2::IS2 lacZ::T7 gene1[plon::IS186] Δ(aaaD-ompT-cusS) Δ(galK-ybhJ) psulA211(-37 T→C) uvrY::IS1::Δ[yecF-dcm-hchA(=yedU)] mcr-73::(mTn10 tetA::IS10) IN(speA210::IS10-xanQ::IS10) endA1(E208K) Δ(yjiT-opgB)114::IS10*	ER2554 X P1(WJ56)--> Pro+ (KanS TetS Lac- T7 RNAP+)	In LCB2	LCB3no2 Gene 1 construction	WJ56 is BL21 *lacZ::gene1*; transduction erased most of LCB3no2 recombination patch

LCB: Locally Co-linear Block; that is, syntenic segments, which have orthologous genes in the same order and orientation. These may suffer large insertions and deletions but not inversions, described more fully in S3 Table Recombination Patches and their Markers

S1 Table

Part B Alleles and Sources

Allele new terminology (old terminology)	centisome	Original strain	Strain source
fhuB478::Tn10 ? (zad::Tn10)	3	RK4374	R. Kadner; *fhuB478::Tn10* is in RK4375
pan-6	3.15-3.21	RK4374	R. Kadner
fhuA2	3.61	NK7254	N. Kleckner
mhpC281::Tn10 (zah-281::Tn10)	7	RS1071	R. Simons via CGSC (#6463)
lacZ::T7 gene1	7.81	WJ56	This work
proC::Tn5	8.71	DR190	D. Roberts
pyrD	21.64	GC4540	S. Gottesman via N. Kleckner
sulA::Tn5	21.98	GC4540	S. Gottesman via N. Kleckner
sulA11	21.98	GW1040	G. Walker via J. Heitman
mcr-73::miniTn10(TetR)	47	ER2475	This work; in ECD_02033 near *thiM*
speA210::Tn10 (zgb-210::Tn10)	61	DF264	D. Fraenkel via CGSC (#6381)
endA1	66.65	MM294	J. Brooks
mcrB1	98.63	LCK8	L. Comai via CGSC (#6515)
opgB(=mdoB)202::Tn10 (zjj202::Tn10)	99	LCK8	L. Comai via CGSC (#6515)
serB28	99.64	PC0950	P.G. de Haan via CGSC (#5409)
Δ(yjiT-opgB)114::IS10 (Δ(mcrC-mrr)114::IS10)	<98.6->98.82	ER1700	Laboratory collection
